# Exosomal miR-19a and IBSP cooperate to induce osteolytic bone metastasis of estrogen receptor-positive breast cancer

**DOI:** 10.1038/s41467-021-25473-y

**Published:** 2021-08-31

**Authors:** Kerui Wu, Jiamei Feng, Feng Lyu, Fei Xing, Sambad Sharma, Yin Liu, Shih-Ying Wu, Dan Zhao, Abhishek Tyagi, Ravindra Pramod Deshpande, Xinhong Pei, Marco Gabril Ruiz, Hiroyuki Takahashi, Shunsuke Tsuzuki, Takahiro Kimura, Yin-yuan Mo, Yusuke Shiozawa, Ravi Singh, Kounosuke Watabe

**Affiliations:** 1 Department of Cancer Biology, Wake Forest University School of Medicine, Winston-Salem, NC USA; 2 Mammary Department, Shuguang Hospital Affiliated to Shanghai University of Traditional Chinese Medicine, Shanghai, China; 3 Department of Breast Surgery, Henan Provincial People’s Hospital, People’s Hospital of Zhengzhou University, People’s Hospital of Henan University, Zhengzhou, Henan China; 4 Department of Breast Surgery, The First Affiliated Hospital of Zhengzhou University, Zhengzhou, Henan China; 5 Department of Pathology, Jikei University School of Medicine, Minato City, Tokyo Japan; 6 Cancer Institute, University of Mississippi Medical Center, Jackson, MS USA

**Keywords:** Cancer, Breast cancer

## Abstract

Bone metastasis is an incurable complication of breast cancer. In advanced stages, patients with estrogen-positive tumors experience a significantly higher incidence of bone metastasis (>87%) compared to estrogen-negative patients (<56%). To understand the mechanism of this bone-tropism of ER^+^ tumor, and to identify liquid biopsy biomarkers for patients with high risk of bone metastasis, the secreted extracellular vesicles and cytokines from bone-tropic breast cancer cells are examined in this study. Both exosomal miR-19a and Integrin-Binding Sialoprotein (IBSP) are found to be significantly upregulated and secreted from bone-tropic ER^+^ breast cancer cells, increasing their levels in the circulation of patients. IBSP is found to attract osteoclast cells and create an osteoclast-enriched environment in the bone, assisting the delivery of exosomal miR-19a to osteoclast to induce osteoclastogenesis. Our findings reveal a mechanism by which ER^+^ breast cancer cells create a microenvironment favorable for colonization in the bone. These two secreted factors can also serve as effective biomarkers for ER^+^ breast cancer to predict their risks of bone metastasis. Furthermore, our screening of a natural compound library identifies chlorogenic acid as a potent inhibitor for IBSP-receptor binding to suppress bone metastasis of ER^+^ tumor, suggesting its preventive use for bone recurrence in ER^+^ patients.

## Abstract (web summary)

Bone metastasis is a major complication of breast cancer (BC) and ER^+^ tumors have a higher incidence of bone metastasis than ER^−^ tumors. Here, the authors report that miR‐19a in exosomes and the bone matrix protein, IBSP, are upregulated and secreted by bone tropic ER^+^ BC cells, where they cooperatively induce osteoclastogenesis and promote bone colonization.

## Introduction

Bone metastasis is the most predominant complication of breast cancer. More than 50% of patients have bone as the first site of distant metastases, followed by lung (17%), brain (16%), and liver (6%)^1^. Ultimately, 70% of metastatic breast cancer patients experience a distant bone relapse during the course of disease^2,3^. Bone metastasis leads to a worse prognosis in breast cancer patients, with a 16-month median survival^4^, and it causes many other complications. Patients develop skeletal-related events (SREs) such as severe bone pain, pathological fracture, hypercalcemia, and infiltration of the bone marrow^5^. Crosstalk between cancer cells and bone stroma cells, including mesenchymal stem cells, osteoblast cells, and osteoclast (OC) cells, play critical roles in bone metastasis^6^. Most breast cancer patients develop osteolytic lesions, which are mainly resulted from OC-mediated bone resorption rather than physical destruction by the cancer cells^7,8^. The interaction with breast cancer cells activates OC cells, induces the bone resorption^9,10^, and liberates the growth factors embedded in the bone matrix, which in turn stimulate the growth of cancer cells^11^. This process is termed “the vicious cycle of bone metastases”^12^. To impede this pathway, bisphosphonate (Zoledronic Acid) and RANKL inhibitor (Denosumab) were developed as bone-targeted therapies^13,14^. However, the use of these drugs in breast cancer patients has several limitations. As to the preventive use of bone-targeted therapies for bone metastasis, while bisphosphonate showed a moderate preventive effect in postmenopausal breast cancer patients, it did not decrease the rate of bone recurrence in premenopausal patients^15^. The significant side effects, including osteonecrosis of the jaws^16^, make it an unfavorable preventive regimen. Moreover, RANKL inhibitor (Denosumab) failed to prevent bone recurrence among both premenopausal and postmenopausal patients^17^. For therapeutic use, these drugs have been proven to reduce the symptoms, but not to extend the patients’ survival^18,19^. Thus, there is an urgent need for effective treatments for preventive and curative management of breast cancer bone metastasis (BCBM).

Among different subtypes of breast cancer, patients with estrogen receptors^+^ (ER^+^) tumors experience the most frequent bone metastases. Bone metastases account for as high as 87% of metastatic diseases among ER^+^ breast cancer patients, while the incidence of bone metastasis is <56% among ER^−^ breast cancer patients^20^ with metastatic diseases. ER positivity is also associated with a higher rate of bone recurrence^21,22^, and ER^+^ breast cancer relapses more frequently in the bone than in other organs^23^. Although ER^+^ breast cancer patients are regularly treated with adjuvant endocrine therapy for a long-term after surgery, cancer cells frequently develop late-onset recurrence in bone^22^. This suggests drug resistance to endocrine therapy developed in the bone-metastatic cells. The long-dormant period also deprioritizes current preventive bone-targeted therapy due to their significant side effects. Thus, it is important to identify biomarkers that can be used for predicting the risk of bone recurrence among ER^+^ breast cancer patients. Despite the strong association between ER positivity and bone metastasis, how ER^+^ cancer cells acquire the bone tropism remains unclear. Understanding the mechanism of such organotropism warrants major opportunities to identify specific biomarkers and preventive/therapeutic targets for ER^+^ breast cancer bone metastasis. To survive in the bone microenvironment, breast cancer cells need to interact with bone microenvironment cells. Cell-secreted free proteins, including PTHrP, IL-1, and IL-6 were known to facilitate the communication between cancer cells and bone cells^24^. In addition to the cytokines, cancer cells are also able to interact with microenvironment cells through extracellular vesicles (EVs)^25^. These vesicles, including apoptotic bodies (ABs), microvesicles (MVs), and exosomes, encapsulate tumor-specific content, and transmit them into environmental cells and circulation. They promote tumor growth by directly transducing tumor cells^26^, or affecting surrounding cells to generate metastatic niche^27–29^. As yet, no secretory factors specific to ER^+^ breast cancer have been identified to elucidate the association between ER positivity and bone metastasis.

In the current study, to decipher the molecular mechanism by which ER^+^ cancer preferentially metastasizes to the bone, we examined both the EVs and the free proteins secreted by ER^+^ bone-tropic breast cancer. We found that the expression of exosomal miR-19a and IBSP is significantly upregulated in the secretion of ER^+^ bone-tropic breast cancer cell lines, as well as in ER^+^ breast cancer patients with bone metastases. While the miR-19a was found to be upregulated in breast cancer, its exact role in tumor progression has been poorly understood^30,31^. IBSP is a major structural protein of the bone matrix and is mainly expressed by bone cells^32^. We found that ectopic expression of both factors promoted bone metastasis in our in vivo models, suggesting both factors cooperate for ER^+^ breast cancer bone metastasis. We also screened a natural compound library and identified chlorogenic acid (CGA) that is capable of blocking this pathway. These findings illustrate a unique pathway of how ER^+^ breast cancer interacts with the bone microenvironment and establishes bone colonization. Furthermore, these two secretory factors would serve as prognostic markers for predicting the risk of bone metastasis in ER^+^ breast cancer patients.

## Results

### Bone-metastatic ER^+^ breast cancer cells secrete miR-19a and IBSP

**Fig. 1:**
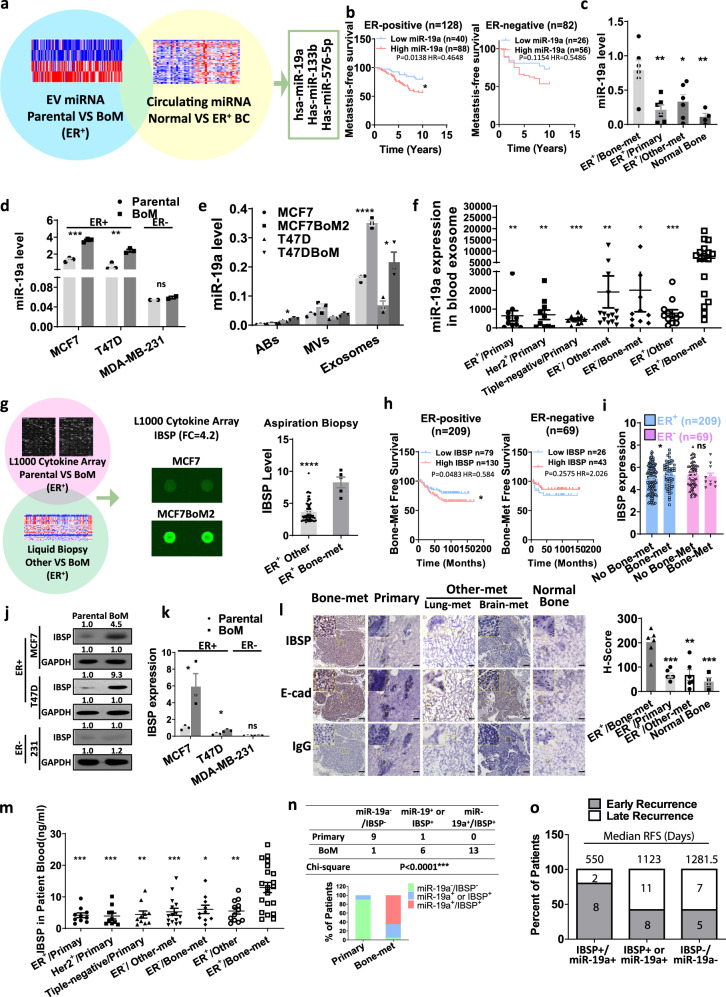
Bone-metastatic ER^+^ breast cancer cells secrete miR-19a and IBSP. **a** EVs were prepared from the conditioned medium of bone-metastatic cell line MCF7BoM2 and the parental cell line MCF7, followed by extraction of RNA. They were then subjected to miRNA profiling by GeneAtlas miRNA Array. Using the criteria of FDR < 0.05 and Fold change > 1.3, 156 miRNAs were selected for further study. These miRNAs were then examined whether they are secreted in the blood of ER^+^ patients using the cohort data set (GSE41922) of the circulating microRNA profiles between 23 ER^+^ breast cancer (BC) patients and 22 healthy donors (Normal). Three miRNAs (miR-19a. miR-133b, miR-576-5p), were found to be significantly upregulated (FDR < 0.05 and logFC > 0.5) in the blood of breast cancer patients. BoM, bone metastatic. **b** Kaplan–Meier analysis of miR-19a was performed between breast cancer patients with low miR-19a and high miR-19a expression using GSE22220. **c** RNA from bone-met(metastatic) lesions (*n* = 6), primary tumors (*n* = 6), lung and brain lesions (*n* = 6) of ER^+^ breast cancer patients, and normal bones (*n* = 4) was extracted and miR-19a expression was examined by Taqman-PCR. A two-sided student’s *t*-test was performed (Primary vs Bone-met, *p* = 0.0047; Other-met vs Bone-met, *p* = 0.0249; Normal Bone vs Bone-met, *p* = 0.0059). Data are presented as mean values ± SEM. **d** The expression of miR-19a in parental cell lines (MCF7, T47D, and MDA-MB-231) and bone-tropic cell lines (MCF7BoM2, T47DBoM, and 231BoM-1833) were assayed by Taqman PCR. Two-sided student’s *t*-tests were performed between parental groups and bone-metastatic (BoM) groups. *p* = 0.0003 (MCF7 vs MCF7BoM2, *n* = 3), *p* = 0.0028 (T47D vs T47DBoM, *n* = 3), *p* = 0.1996 (MDA-MB-231 vs 231BoM-1833, *n* = 2). Data are presented as mean values ± SEM. **e** The expressions of miR-19a in ABs, MVs, and exosomes from the parental cell lines (MCF7 and T47D) as well as their bone-tropic variants (MCF7BoM2 and T47DBoM) were examined by Taqman PCR. Two-sided student’s *t*-tests were performed between parental groups and bone-metastatic (BoM) groups, *n* = 3 in each group. *p* = 0.0408 (ABs, T47D vs T47DBoM), *p* < 0.0001 (Exosomes, MCF7 vs MCF7BoM2), *p* = 0.0167 0001 (Exosomes, T47D vs T47DBoM). Data are presented as mean values ± SEM. **f** Exosomes were isolated from the serum of breast cancer patients and the expression of miR-19a was compared among seven groups: (i) ER^+^ (*n* = 10), (ii) Her2^+^ (*n* = 10), and (iii) triple-negative (*n* = 10) breast cancer patients who were recurrence-free for 10 years, and (iv) ER^−^ breast cancer patients with visceral metastases (*n* = 15), (v) ER^−^ breast cancer patients with bone metastasis (*n* = 10), (vi) ER^+^ breast cancer patients with visceral metastases (*n* = 12) and ER^+^ breast cancer patients with bone metastasis (*n* = 20). Two-sided student’s *t*-tests were performed to compare the miR-19a expression between the ER^+^/Bone-met group with each of the other groups. *p* = 0.0012, 0.003, 0.0008, 0.0036, 0.016, and 0.0005 from left to right. Data are presented as mean values ± SEM. **g** Cytokine/growth factor profiling was performed on conditioned media from MCF7 and MCF7BoM2 using L1000 array (Raybiotech), and 49 upregulated cytokines (fold change > 3) were selected. This list was cross-examined with gene profiles of the ER^+^ breast cancer biopsy database (GSE56493). Genes upregulated (FDR < 0.05 and fold change > 1.2) in biopsy of bone metastasis (*n* = 5) compared to other sites (*n* = 75) were selected. IBSP in conditioned media, as well as in ER^+^ breast cancer biopsy from the bone lesion and other sites (GSE56493, *p* < 0.0001, presented as mean values ± SEM) were presented. **h** Kaplan–Meier analyses were performed for the association between IBSP expression and bone metastasis-free survival among 209 ER^+^ breast cancer patients and 69 ER^−^ breast cancer patients using GSE2034. **i** The IBSP expression was compared between ER^+^ breast cancer patients with (n = 57) or without (*n* = 152) bone metastasis (*p* = 0.04625, two-sided student’s *t*-test), as well as between ER^−^ breast cancer patients with (*n* = 11) or without (*n* = 58) bone metastases (*p* = 0.6944, two-sided student’s *t*-test). Data are presented as mean values ± SEM. **j** Western blot analysis for IBSP expression in MCF7 and MCF7BoM2, T47D and T47DBoM, MDA-MB-231 and 231BoM-1833. Quantification of the bands was performed using the ImageJ program and the value was normalized to that of control (left panel). **k** PCR analysis for IBSP expression was performed in MCF7 and MCF7BoM2 (*p* = 0.0341), T47D and T47DBoM (*p* = 0.0120), MDA-MB-231 and 231BoM-1833 (*p* = 0.3494), *n* = 3 in all groups. Two-sided student’s *t*-tests were used in each pair of comparisons. Data are presented as mean values ± SEM. **l** IBSP was examined by IHC on bone lesions (*n* = 6), primary breast cancer tissue without recurrence for 10 years (*n* = 6), brain and lung lesions (*n* = 6) and normal bones (*n* = 4). The staining was quantified by *H*-score assessment and compared by the two-sided student’s *t*-test. *p* = 0.0002 (Primary vs Bone-met); *p* = 0.0014 (Other-met vs Bone-met); *p* = 0.0006 (Normal Bone vs Bone-met). Data are presented as mean values ± SEM. E-cad (E-cadherin) was stained as the marker of breast cancer cells. Rabbit IgG was used as isotype control. Scale bar, 100 µm. **m** ELISA was performed to quantify the IBSP level in the serum of ER^+^ (*n* = 10), Her2^+^ (n = 10) and triple-negative (*n* = 10) breast cancer patients without recurrence for 10 years, and ER^−^ breast cancer patients with visceral metastases (*n* = 15), ER^−^ breast cancer patients with bone metastasis (*n* = 10), ER^+^ breast cancer patients with visceral metastases (*n* = 12) and ER^+^ breast cancer patients with bone metastasis (*n* = 20). Two-sided student’s *t*-tests were performed to compare the miR-19a expression between the ER^+^/Bone-met group with each of the other groups. *p* = 0.0009, 0.0009, 0.0016, 0.0010, 0.0101, and 0.0024 from left to right. Data are presented as mean values ± SEM. **n** ER^+^ breast cancer patients with or without bone metastases were separated into three groups according to the serum levels of IBSP and exosomal miR-19a, including miR-19a low/IBSP low group (miR-19a^−^/IBSP^−^), miR-19a low/IBSP high or miR-19a high/IBSP low group (miR-19^+^ or IBSP^+^) and miR-19a high/IBSP high group (miR-19a^+^/IBSP^+^). The percentage in each group was calculated and plotted as the bar graph. The chi-square test was performed. **o** ER^+^ breast cancer patients with recurrent disease were selected from TCGA. Patients with early recurrence (<1000 days) and late recurrence (≥1000 days) were separated into three groups: miR-19a low/IBSP low group (miR-19a^−^/IBSP^−^), miR-19a low/IBSP high or miR-19a high/IBSP low group (miR-19^+^ or IBSP^+^) and miR-19a high/IBSP high group (miR-19a^+^/IBSP^+^). The percentage of patients and the median RFS (recurrence-free survival) days in each group were calculated.

Considering the importance of cell–cell communication in bone metastasis, ER^+^ cells may shed secretory factors in the bone microenvironment to exert strong bone tropism. To test this hypothesis, we examined both EVs and proteins secreted from bone-tropic breast cancer cells. Small non-coding RNA is known to be selectively transported and enriched in EVs^33,34^, suggesting its importance as a disease regulator and feasible biomarker. Thus, we compared the transcriptome profiles of miRNA in EVs isolated from MCF7BoM2, a previously reported organotropic ER^+^ breast cancer cell line that prefers bone colonization^35^, with the EVs from its parental cell line MCF7 (Fig. 1a). We made sure that the increased bone metastasis rate of the MCF7BoM2 line was not due to an alteration in the cell growth ability, as implantation of MCF7 and MCF7BoM2 into the mammary fat pads generated primary tumors with similar sizes and weights (Supplementary Fig. 1a–d). We found 157 miRNAs that were significantly upregulated in the EVs from MCF7BoM2. To validate the clinical relevance of these exosomal miRNAs, we examined whether they are secreted into the blood of ER^+^ patients using the cohort data set of the circulating miRNA profiles from 23 ER^+^ breast cancer patients and 22 healthy donors (Fig. 1a). Among the 157 miRNAs, three of them (miR-19a, miR-133b, and miR-576-5p) were found to be significantly upregulated in the blood of ER^+^ breast cancer patients. Kaplan–Meier survival analyses were performed to investigate the association between these three miRNAs with metastatic events, and only miR-19a was found to be positively correlated with metastatic disease in ER^+^ breast cancer patients (Fig. 1b). There is no difference in metastatic incidence between ER^−^ breast cancer patients with or without elevated miR-19a expression (Fig. 1b), and both miR-133b and miR-576-5p are not significantly associated with metastasis in either ER^+^ or ER^−^ patients (Supplementary Fig. 2a). We also compared the ER^+^ breast cancer tissue from bone-metastatic lesions with ER^+^ primary breast cancer tissue from breast cancer patients without recurrence for 10 years, and with brain and lung metastatic lesions from ER^+^ breast cancer patients with visceral metastases only, as well as with normal bone tissue. It was found that miR-19a is indeed only increased in bone-metastatic tissues (Fig. 1c). To validate the specificity of miR-19a associated with ER^+^ tumor, we established another ER^+^ bone-tropic cell line, T47DBoM. Similar to MCF7BoM2, T47DBoM generated comparable size and weight of primary tumors when implanted in the mammary fat pads. On the other hand, T47DBoM caused more aggressive bone metastases when it was implanted intravenously (Supplementary Fig. 1e–h). We examined the expression of miR-19a in ER^+^ and ER^−^ cancer cell lines and found that the ER^+^ bone-tropic breast cancer cells, MCF7BoM2 and T47DBoM, have a higher endogenous expression of miR-19a compared to the parental cell lines, MCF7 and T47D (Fig. 1d). On the other hand, the expression of miR-19a did not show any difference in the bone-tropic triple-negative cell line derived from MDA-MB-231^36^ (Fig. 1d). To examine whether free or vesicle-encapsulated miRNAs are the major source of secreted miR-19a, we treated the conditioned medium from MCF7BoM2 with RNase. The RNase treatment did not degrade the secreted miR-19a from breast cancer cells, suggesting that they are protected in the membrane vesicles (Supplementary Fig. 2b). This finding is consistent with the previous report that the majority of miRNA in serum is encapsulated by EVs^37,38^. EVs include ABs, MVs and exosomes. To further clarify the source of EV-derived miR-19a, we examined the miR-19a expression in these three components of EVs from MCF7 and T47D as well as their bone-tropic variants. After confirming the purity of the isolated vesicles by nanoparticle tracking analysis (NTA), electron microscope (EM), as well as a western blot (Supplementary Fig. 2c, d), the expression of miR-19a in these vesicles, were examined by Taqman PCR. We found that miR-19a is most highly expressed in exosomes compared to other EVs (Fig. 1e). Importantly, there is a strong upregulation of miR-19a in the exosomes from ER^+^ bone-metastatic variants, MCF7BoM2 and T47DBoM (Fig. 1e), compared to their parental cell lines. However, ER^−^ 231BoM showed only a negligible increase of miR-19a in the exosomes (Supplementary Fig. 2e). Next, we examined whether the increase of exosomal miR-19a can be detected in the serum of ER^+^ breast cancer patients with bone metastases. Strikingly, ER^+^ breast cancer patients with bone metastasis are significantly associated with higher expression of exosomal miR-19a in serum (Fig. 1f). It should be noted that the expression of exosomal miR-19a remained low in the patients with primary breast cancer with no recurrence within 10 years. We also did not observe any increase in exosomal miR-19a in the serum of ER^−^ breast cancer patients at advanced stages nor in ER^+^ patients with visceral metastases but with no bone involvement (Fig. 1f). These data suggest that miR-19a is specifically upregulated in both endogenous and exosomal levels of ER^+^ bone-tropic breast cancer cells.

We also examined secreted proteins and cytokines from ER^+^ bone-tropic breast cancer cells. By performing L1000 cytokine array analysis, 1000 secreted proteins were examined in the conditioned medium from MCF7 and MCF7BoM2, and 49 proteins were found to be upregulated in the cell culture media from MCF7BoM2. Among these 49 proteins, IBSP was the only significantly upregulated gene in the aspiration biopsy from bone-metastatic lesions of ER^+^ breast cancer patients, compared to the biopsy samples from other sites (Fig. 1g). IBSP is a major structural protein of the bone matrix. It is synthesized by skeletal-associated cell types as a secreted protein, and is able to bind to calcium and hydroxyapatite via its acidic amino acid clusters^39^. In breast cancer, we observed cancer cell-specific upregulation of IBSP, while the paired normal breast epithelial cells did not express IBSP (Supplementary Fig. 2f). This finding suggests a possible role of IBSP in breast cancer progression. Since many cytokines were previously reported to bind to the surface of EVs, we also looked at the membrane fractions of EVs by western blot (Supplementary Fig. 2g), but IBSP was not detected. We also performed immunostaining of IBSP and CD63 for the exosomes isolated from MCF7BoM2, and found that only CD63 but not IBSP was detected on the surface of exosomes (Supplementary Fig. 2h). Thus, we concluded that IBSP in the extracellular region is mainly in the form of free secreted protein. To further clarify the connection between IBSP and bone metastasis of ER^+^ breast cancer, we performed Kaplan–Meier analyses among 278 breast cancer patients. IBSP was found to be positively correlated with bone metastasis only in ER^+^ breast cancer patients, but not in ER^-^ breast cancer patients (Fig. 1h). There is an increase in IBSP expression in the bone-metastatic group compared to the non-bone-metastatic group among ER^+^ breast cancer patients, but not among ER^−^ breast cancer patients (Fig. 1i). We also verified that ER^+^ bone-metastatic cell lines have higher expression of IBSP compared to the parental cell lines. The PCR and western blot analyses confirmed the upregulation of IBSP in MCF7BoM2 and T47DBoM, but not in ER^−^ bone-metastatic cell line 231BoM (Fig. 1j, k). When ER^+^ breast cancer tissue from bone lesions was compared with ER^+^ primary breast cancer tissue from patients with no recurrence within 10 years, and with brain and lung metastatic lesions from ER^+^ breast cancer patients with visceral metastases only, as well as with normal bone tissue, IBSP was found to be significantly increased in bone-metastatic tissues (Fig. 1l). Next, we examined whether the increase of IBSP can be detected in the serum of ER^+^ breast cancer patients with bone metastases. A significant upregulation of IBSP protein in the serum of ER^+^ breast cancer patients with bone metastasis was detected (Fig. 1m), but not in the serum of patients with only primary breast cancer, advanced ER^-^ breast cancer patients, or ER^+^ breast cancer patients with visceral metastases other than bone.

Our data (Fig. 1a–m) suggest both miR-19a and IBSP are positively related to bone metastasis of ER^+^ cancer cells. However, how they are associated with bone metastasis remains unknown. We further investigated whether miR-19a and IBSP are mutually related to bone metastasis of ER^+^ breast cancer cells. We combined the results from Fig. 1f with Fig. 1m, and stratified the patients into three groups: both miR-19a and IBSP low, miR-19a or IBSP high, both miR-19a and IBSP high. Strikingly, ER^+^ breast cancer patients with bone metastasis are significantly associated with higher expression of both IBSP and miR-19a. Upregulation of sole factor led to a negligible, but not significant increase of the bone-metastatic incidence, suggesting a possible cooperative role of these two factors in bone metastasis of ER^+^ breast cancer cells (Fig. 1n). Next, we examined the expression of miR-19a and IBSP in breast cancer patients using the TCGA database. Among 41 ER^+^ breast cancer patients with recurrence, IBSP and miR-19a could predict the recurrence risk as the majority of patients with high levels of both factors experienced earlier recurrence within 1000 days (Fig. 1o). The median recurrence-free survival (RFS) time of patients with high levels of both miR-19a and IBSP is only 550 days, while the median RFS time of patients with high expression of either miR-19a or IBSP and patients with low levels of both miR-19a/IBSP are 1123 days and 1281.5 days, respectively. These results indicate that miR-19a and IBSP are positively associated with bone metastasis, and they serve as biomarkers for a higher risk of bone metastasis when they are both upregulated in ER^+^ breast cancers.

### Both IBSP and miR-19a are essential for bone metastasis of ER^+^ breast cancer

**Fig. 2:**
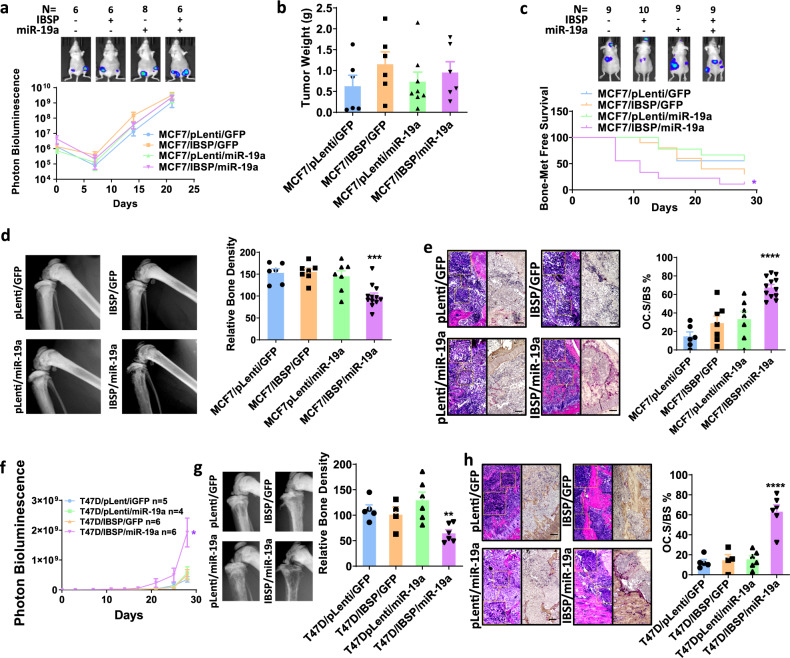
Both IBSP and miR-19a are essential for bone metastasis of ER^+^ breast cancer. **a** miR-19a and/or IBSP were ectopically expressed in MCF7, and they were transplanted into mammary fat pads of female nude mice. The growth of the tumor was monitored by measuring the luciferase activity by IVIS Bioimager. *p* = 0.3110 (MCF7/pLenti/GFP vs MCF7/IBSP/miR-19a; *n* = 6 vs 6), *p* = 0.2649 (MCF7/pLenti/GFP vs MCF7/IBSP/GFP; *n* = 6 vs 6), *p* = 0.4739 (MCF7/pLenti/GFP vs MCF7/pLenti/miR-19a; *n* = 6 vs 8). Data are presented as mean values ± SEM. **b** At day 21, tumors were removed from the mammary fat pad and their weights were measured. *p* = 0.3971 (MCF7/pLenti/GFP vs MCF7/IBSP/miR-19a; *n* = 6 vs 6), *p* = 0.2178 (MCF7/pLenti/GFP vs MCF7/IBSP/GFP; *n* = 6 vs 6), *p* = 0.7632 (MCF7/pLenti/GFP vs MCF7/pLenti/miR-19a; *n* = 6 vs 8). Data are presented as mean values ± SEM. **c** miR-19a and/or IBSP were ectopically expressed, and they were transplanted into female nude mice via intracardiac injection. The incidence of bone metastasis was monitored by measuring the luciferase activity by IVIS Bioimager. Log-rank (Mantel–Cox) test was performed to calculate the *p*-value. *p* = 0.0105 (MCF7/pLenti/GFP vs MCF7/IBSP/miR-19a), *p* = 0.3982 (MCF7/pLenti/GFP vs MCF7/IBSP/GFP), *p* = 0.9230 (MCF7/pLenti/GFP vs MCF7/pLenti/miR-19a). **d** The legs of mice were imaged by X-ray and the bone density was measured by ImageJ. *p* = 0.0007 (MCF7/pLenti/GFP vs MCF7/IBSP/miR-19a; *n* = 6 vs 12), *p* = 0.8621 (MCF7/pLenti/GFP vs MCF7/IBSP/GFP, *n* = 6 vs 7), *p* = 0.6397 (MCF7/pLenti/GFP vs MCF7/pLenti/miR-19a, *n* = 6 vs 7). Two-sided student’s *t*-tests were performed. Data are presented as mean values ± SEM. **e** TRAP staining was performed in tumor-bearing bones from mice. The total OC surface area relative to the bone surface area was measured by ImageJ, then calculated and compared among different groups. H&E staining of the same field was shown together with the TRAP staining. Scale bar, 100 µm. *p* < 0.0001 (MCF7/pLenti/GFP vs MCF7/IBSP/miR-19a, *n* = 6 vs 12), *p* = 0.1598 (MCF7/pLenti/GFP vs MCF7/IBSP/GFP, *n* = 6 vs 7), *p* = 0.0867(MCF7/pLenti/GFP vs MCF7/pLenti/miR-19a, *n* = 6 vs 7). Two-sided student’s *t*-tests were performed. Data are presented as mean values ± SEM. **f** miR-19a and/or IBSP were ectopically expressed, and they were transplanted into female nude mice via intra-tibia injection. The growth of tumor in bone was monitored by measuring the luciferase activity by IVIS Bioimager. *p* = 0.0175 (T47D/pLenti/GFP vs T47D/IBSP/miR-19a), *p* = 0.4877 (T47D/pLenti/GFP vs T47D/IBSP/GFP), *p* = 0.3305 (T47D/pLenti/GFP vs T47D/pLenti/miR-19a). Data are presented as mean values ± SEM. **g** The legs of mice were imaged by X-ray and the bone density was measured by ImageJ. *p* = 0.0034 (T47D/pLenti/GFP vs T47D/IBSP/miR-19a *n* = 5 vs 6), *p* = 0.5750 (T47D/pLenti/GFP vs T47D/IBSP/GFP, *n* = 5 vs 4), *p* = 0.3809 (T47D/pLenti/GFP vs T47D/pLenti/miR-19a, *n* = 5 vs 6). Two-sided student’s *t*-tests were performed. Data are presented as mean values ± SEM. **h** TRAP staining was performed in tumor-bearing bones from the mice. The OC surface relative to the bone surface was calculated and compared among different groups. H&E staining of the same field was shown together with the TRAP staining. Scale bar, 100 µm. *p* = 0.0001 (T47D/pLenti/GFP vs T47D/IBSP/miR-19a, *n* = 5 vs 6), *p* = 0.7711 (T47D/pLenti/GFP vs T47D/IBSP/GFP, *n* = 5 vs 4), *p* = 0.5701 (T47D/pLenti/GFP vs T47D/pLenti/miR-19a, n = 5 vs 6). Two-sided student’s *t*-tests were performed. Data are presented as mean values ± SEM.

To test whether both miR-19a and IBSP are required for bone metastasis of ER^+^ breast cancer cells, we ectopically expressed IBSP and miR-19a in MCF7, and established stable overexpression cell lines (Supplementary Fig. 3a–d). Ectopic expression of miR-19a did not alter the level of IBSP, and vice versa, indicating there is no regulatory network involved between IBSP and miR-19a. Neither miR-19a nor IBSP could induce any change of exosome production (Supplementary Fig. 3e), proliferation, or migration of the cells in vitro (Supplementary Fig. 3f, g). Intra-mammary fat pad injection of MCF7 with miR-19a and/or IBSP forced-expression did not induce any difference in tumor growth in vivo (Fig. 2a, b). Strikingly, ectopic expression of both miR-19a and IBSP together in MCF7 led to a significant increase in bone metastasis in the xenograft model with the intracardiac injection of cancer cells (Fig. 2c). Nonetheless, miR-19a or IBSP alone did not increase the incidence of bone metastasis. These data suggest miR-19a and IBSP promote bone metastasis through the tumor microenvironment instead of affect cancer cells directly. Since IBSP is known to regulate bone turnover by binding to integrin αVβ3 receptor on the surface of OC cells^40^, we examined the bone densities of mice implanted with the breast cancer cells. We found that ectopic expression of both IBSP/miR-19a resulted in a significant decrease in bone density in the tumor lesions (Fig. 2d). We also quantified the total area of TRAP ^+^ OC surface and its percentage of total bone surface area (OC.S/BS%) in the tumor-bearing bones and found that synchronous overexpression of miR-19a and IBSP significantly increased the osteoclastogenesis (Fig. 2e). We then repeated this experiment in another ER^+^ cell line, T47D. Both IBSP and miR-19a were ectopically introduced into T47D (Supplementary Fig. 3h, i), and stable overexpression cell lines had been established (Supplementary Fig. 3j, k) before the cells were inoculated into mouse tibia. Similar to MCF7, ectopic expression of both miR-19a and IBSP resulted in increased tumor burden in the bones (Fig. 2f), while discrete expression of miR-19a or IBSP alone did not affect the tumor growth. Bone lesions of T47D/IBSP/miR-19a presented decreased bone densities (Fig. 2g) and increased OC activities (Fig. 2h). These results indicate that miR-19a and IBSP work together to induce osteolytic bone metastasis in ER^+^ breast cancer patients, though not directly affecting cancer cell growth or motility.

### miR-19a promotes osteolytic bone metastasis

**Fig. 3:**
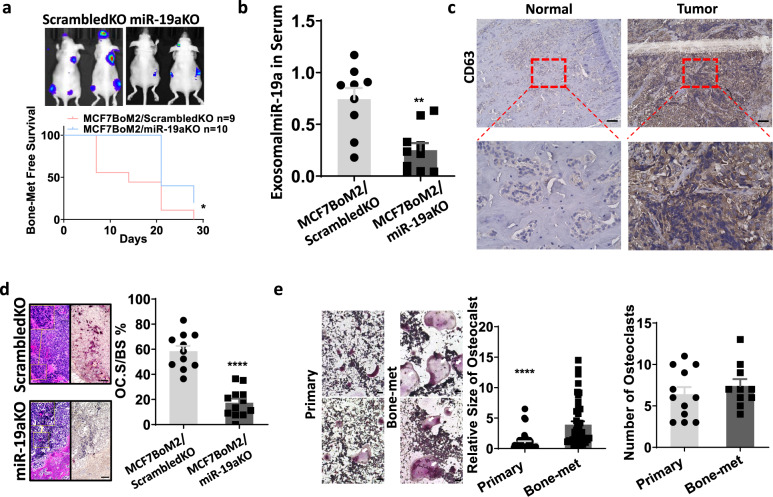
miR-19a promotes osteolytic bone metastasis. **a** MCF7BoM2/ScrambledKO and MCF7BoM2/miR-19aKO were transplanted into female nude mice via intracardiac injection. The growth of bone metastasis was monitored by measuring the luciferase activity by IVIS Bioimager. Log-rank (Mantel–Cox) test was performed to calculate the *p*-value (*p* = 0.0109). **b** At the endpoint (day 28), mice were sacrificed and the expression of exosomal miR-19a from serum was examined by Taqman. A two-sided student’s *t*-test was performed (*p* = 0.0011, *n* = 9 vs 10). Data are presented as mean values ± SEM. **c** Representative figures of the expression of exosomal marker CD63 in normal bone and tumor-bearing bone from the mice. Scale bar, 100 µm. **d** TRAP staining for osteoclast cells in tumor-bearing bones from the mice transplanted with MCF7BoM2/ScrambledKO or MCF7BoM2/miR-19aKO. OC surface relative to the bone surface was calculated and compared. H&E staining of the same field was shown together with the TRAP staining. Scale bar, 100 µm. A two-sided student’s *t*-test was performed (*p* = 0.00000014, *n* = 11 vs 12). Data are presented as mean values ± SEM. **e** Exosomes from the serum of ER^+^ breast cancer patients with (*n* = 10) or without (*n* = 12) bone metastasis were isolated. The harvested exosomes were incubated with mouse bone marrow-derived monocytes. After 1 week, the cells were fixed and TRAP staining was performed. The size (*p* = 0.0000039) and number (*p* = 0.4254) of differentiated OC were quantified and compared. Scale bar, 100 µm. Two-sided student’s *t*-tests were performed. Data are presented as mean values ± SEM.

Because both miR-19a and IBSP are necessary to promote the bone metastasis of ER^+^ breast cancer cells, we investigated how each of them contributes to the bone-tropic metastasis of cancer cells. MiR-19a, as a member of the OncomiR-1 family, was previously found to be upregulated in multiple malignancies^41–43^. It was also found to be expressed in breast cancer cells, although its role remains unclear^30,44^. We found both endogenous and secreted levels of miR-19a were higher in the bone-metastatic cell lines compared to the parental cell lines. However, endogenous expression of miR-19a did not affect breast cancer cells per se because the ectopic expression of miR-19a did not alter the growth or motility of cells in vitro (Supplementary Fig. 3f, g). To further clarify the role of miR-19a in bone metastasis of ER^+^ breast cancer cells, we applied the CRISPR/Cas9 technology to knock out the miR-19a in MCF7BoM cells. A specific gRNA (guide RNA) was designed to target the stem-loop of miR-19a precursor so that the biogenesis of mature miR-19a was hindered. The genomic DNA cleaving ability of our gRNA targeting miR-19a was verified by the T7E1 enzyme (Supplementary Fig. 4a). Four independent clones with minimal miR-19a expression and unaltered proliferation were selected and combined to establish the stable knockout cell line (Supplementary Fig. 4b–d). When tested in vitro, we found that knockout of miR-19a did not alter the cell migration or invasion abilities (Supplementary Fig. 4e–f). However, when miR-19a knockout cells were implanted into nude mice by intracardiac injection, loss of miR-19a significantly decreased bone metastasis (Fig. 3a). These data are consistent with our previous finding that miR-19a is needed for bone-tropic metastasis of MCF7BoM2. MCF7BoM2 has a high expression of both miR-19a and IBSP. Knockout of miR-19a, but leaving IBSP alone, compromised the bone-metastatic ability of MCF7BoM2. Interestingly, when we examined the serum exosomes in the mice, we found that the knockout of miR-19a significantly decreased exosomal miR-19a in the serum (Fig. 3b). This finding suggests that serum exosome could represent the dynamic changes of breast cancer cells. These results are also in agreement with the previous finding that miR-19a could not induce the intrinsic change of cancer motility. Accordingly, we investigated whether exosomal miR-19a could promote bone metastasis by remodeling environmental cells in a paracrine manner instead of directly affecting cancer cells. The tumor lesion in the bone exhibited abundant expression of exosomal marker CD63 (Fig. 3c), implying exosome activity in the microenvironment. Since we found an aberrant osteolytic change in our previous data, we examined the OC in the bone lesions. There was a decrease of osteoclastogenesis in the tumor-bearing bones when miR-19a was knocked out from the cancer cells (Fig. 3d), indicating a possible role of exosomal miR-19a in promoting OC cells. To verify this hypothesis, exosomes from serum of ER^+^ breast cancer patients with or without bone metastases were isolated and incubated with primary OC precursors. Exosomes prepared from the patients with bone metastases significantly promoted OC maturation and differentiation (Fig. 3e). These results indicate that miR-19a is involved in the osteolytic bone metastasis and exosomal miR-19a could directly promote OC activity in the bone microenvironment.

### Exosomal miR-19a targets PTEN/AKT pathway to promote osteoclastogenesis

**Fig. 4:**
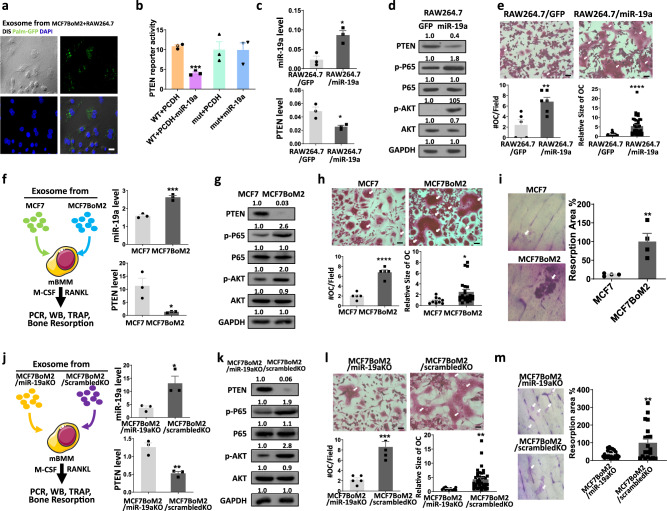
Exosomal miR-19a targets PTEN/AKT pathway to promote osteoclastogenesis. **a** Exosomes were labeled with GFP by ectopic expression of PalmGFP in MCF7BoM2 cells. OC cells were then treated with the labeled exosomes. After 24 h, uptake of exosome by osteoclast cells was monitored by confocal microscope. Scale bar, 10 µm. **b** Luciferase reporter assay for the constructs containing wild-type PTEN 3′UTR or mutant PTEN 3′UTR was performed after transfecting the cells with the miR-19a expression vector. *p* = 0.0001 (WT + PCDH vs WT + PCDH-miR-19a, two-sided student’s *t*-test). Data are presented as mean values ± SEM. **c** Taqman PCR for miR-19a expression (*p* = 0.0131, *n* = 3), and SYBR Green qPCR for PTEN expression (*p* = 0.0232, *n* = 3) in RAW264.7/GFP and RAW264.7/miR-19a were performed. Two-sided student’s *t*-tests were performed. Data are presented as mean values ± SEM. **d** Western blot analysis for PTEN, phospho-NF-κB P65, total NF-κB P65, phospho-AKT and total AKT expression in RAW264.7/GFP and RAW264.7/miR-19a cell lines. The band intensity was quantified and normalized to that of control (left panels). **e** TRAP staining of RAW264.7/GFP and RAW264.7/miR-19a cells. On day 4, the number of differentiated osteoclasts (*p* = 0.0080, *n* = 5 vs 6) and the size of these cells (*p* < 0.0001, *n* = 30 vs 40) were measured. Two-sided student’s *t*-tests were performed. Data are presented as mean values ± SEM. Scale bar, 100 µm. **f** Mouse BMMs were induced into OC cells with the treatment of exosomes and cytokines. Taqman PCR for miR-19a expression (*p* = 0.0008, *n* = 3) and SYBR Green qPCR for PTEN (*p* = 0.0271, *n* = 3) expression were performed for OC cells treated with exosomes from MCF7 and MCF7BoM2. Two-sided student’s *t*-tests were performed. Data are presented as mean values ± SEM. **g** Western blot analysis for PTEN, phospho-NF-κB P65, total NF-κB P65, phospho-AKT and total AKT expression in mouse BMMs treated with exosomes from MCF7 and MCF7BoM2. The band intensity was quantified and normalized to that of control (left panels). **h** TRAP staining was performed for mouse BMMs treated with exosomes from MCF7 and MCF7BoM2. On day 4, the number of differentiated osteoclast (*p* = 0.000036, *n* = 5 vs 5) and their sizes (*p* = 0.0323, *n* = 10 vs 23) were measured. Two-sided student’s *t*-tests were performed. Data are presented as mean values ± SEM. Scale bar, 100 µm. **i** Bone-resorbing activities of OC treated with exosomes from MCF7 and MCF7BoM2 were measured by the bone-resorption assay. BMMs were seeded on a bone chip and treated with exosomes and cytokines. Resorption pits were measured and compared (*p* = 0.0070, *n* = 4). Two-sided student’s *t*-tests were performed. Data are presented as mean values ± SEM. **j** Taqman PCR for miR-19a expression (*p* = 0.0291, *n* = 3) and SYBR Green qPCR for PTEN expression (*p* = 0.0047, *n* = 3) were performed for mouse BMMs treated with exosomes from MCF7BoM2/miR-19aKO and MCF7BoM2/ScrambledKO. Two-sided student’s *t*-tests were performed. Data are presented as mean values ± SEM. **k** Western blot analysis was performed for PTEN, phospho-NF-κB P65, total NF-κB P65, phospho-AKT, and total AKT expression in mouse BMMs treated with exosomes from MCF7BoM2/miR-19aKO and MCF7BoM2/ScrambledKO. The band intensity was quantified and normalized to that of control (left panels). **l** TRAP staining was performed for mouse BMMs that were treated with exosomes from MCF7BoM2/miR-19aKO and MCF7BoM2 /ScrambledKO. On day 4, the number of differentiated osteoclast cells (*p* = 0.0005, *n* = 5 vs 5) and their sizes were measured (*p* = 0.0029, *n* = 11 vs 38). Scale bar, 100 µm. Two-sided student’s *t*-tests were performed. Data are presented as mean values ± SEM. **m** The bone-resorbing activity of OC was measured by the bone-resorption assay using BMMs that were treated with exosomes from MCF7BoM2/miR-19aKO and MCF7BoM2/ScrambledKO (*p* = 0.0025, *n* = 24 vs 20). Two-sided student’s *t*-tests were performed. Data are presented as mean values ± SEM.

To validate our hypothesis that exosomal miR-19a promotes the activity of OC cells, we first examined whether exosomes can be directly up-taken by OC cells. OC precursor RAW264.7 cells were treated with PalmGFP-labeled exosomes derived from bone-metastatic breast cancer cells. The result indicates that exosomes from MCF7BoM2 were indeed incorporated into RAW264.7 cells 24 h after the treatment (Fig. 4a). It was reported that miR-19a targeted PTEN 3′UTR^45^ and that OC differentiation and their activity were regulated by NF-κB and AKT pathways^46,47^, both of which were known to be inhibited by PTEN^48,49^. Thus, it is plausible that cancer cells secrete miR-19a and suppress PTEN expression in OC cells when exosomes are internalized. The decrease of PTEN leads to activation of NF-κB and AKT pathways that promote OC cells. To test this notion, we examined whether exosomal miR-19a modulates OC activity by suppressing PTEN expression, using the 3′UTR reporter assay. We confirmed that the miR-19a-targeted sequence on PTEN is conserved between human and mouse (Supplementary Fig. 5a, b), and found that ectopic expression of miR-19a indeed targeted PTEN, while mutation of the miR-19a binding site on PTEN 3′UTR rescued the reporter activity (Fig. 4b). Furthermore, ectopic expression of miR-19a in OC precursor cells, RAW264.7, suppressed PTEN expression and induced NF-κB and AKT pathway with increased phosphorylated P65 and AKT (Fig. 4c, d). We also examined whether increased miR-19a could promote OC differentiation of RAW264.7 by performing TRAP staining. The ectopic expression of miR-19a significantly increased the number of mature OC cells and their size (Fig. 4e). Next, we tested whether direct treatment of OC precursor cells with exosomal miR-19a, instead of lentiviral overexpression, could also induce the activation of P65 and AKT pathways. Although there was no difference in the efficiency of exosome uptake (Supplementary Fig. 5c), compared to exosomes from the parental cell line, exosomes from bone-metastatic MCF7BoM2 increased miR-19a, decreased PTEN and induced AKT, and P65 activation in mouse bone marrow monocyte (mBMM), which is the primary precursor of OC (Fig. 4f, g). In addition, exosomes from MCF7BoM2 promoted the differentiation of mBMM into OC (Fig. 4h). In a bone resorption assay, mBMMs were seeded on the bone chips and incubated with exosomes from MCF7 and MCF7BoM2. Exosomes from MCF7BoM2 exhibited significantly higher competence to induce the osteolytic activities of OC cells as shown by the increased area of resorption pit (Fig. 4i). Consistent with our finding of enriched miR-19a in exosomes, only exosomes, but not MV or AB, could induce the differentiation of OC cells (Supplementary Fig. 5d). To further investigate whether miR-19a is responsible for the OC-promoting effect of the exosomes, OC cells were incubated with exosomes isolated from MCF7BoM2/ScrabmbleKO cells or MCF7BoM2/miR-19aKO cells (Fig. 4j–m and Supplementary Fig. 6a–c). When miR-19a was knockout, exosomes were unable to deliver miR-19a into OC cells and suppress PTEN expression (Fig. 4j and Supplementary Fig. 6b). We also found that exosomes prepared from bone-metastatic breast cells with miR-19a knockout could not induce P65 and AKT activation (Fig. 4k and Supplementary Fig. 6c) and reversed the OC-promoting effect (Fig. 4l, m and Supplementary Fig. 6a). Collectively, we demonstrated that bone-metastatic cells delivered miR-19a-containing exosomes to OC precursor cells and suppressed PTEN expression. Decreased PTEN induced the activation of NF-κB and AKT signaling pathways, promoting the differentiation and osteolytic activity of OC cells. This notion was also verified by examining the effect of exosomes from MCF7 and MCF7/miR-19a cell lines on OC cells. Compared to exosomes from parental MCF7 cells, exosomes from MCF7 with ectopic expression of miR-19a significantly increased the miR-19a expression and decreased PTEN expression in both recipient RAW264.7 and mBMM cells, increased OC number, and promoted the differentiation of OC in vitro (Supplementary Fig. 6d–i).

### ER^+^ breast cancer cells secrete IBSP to recruit osteoclast precursors

**Fig. 5:**
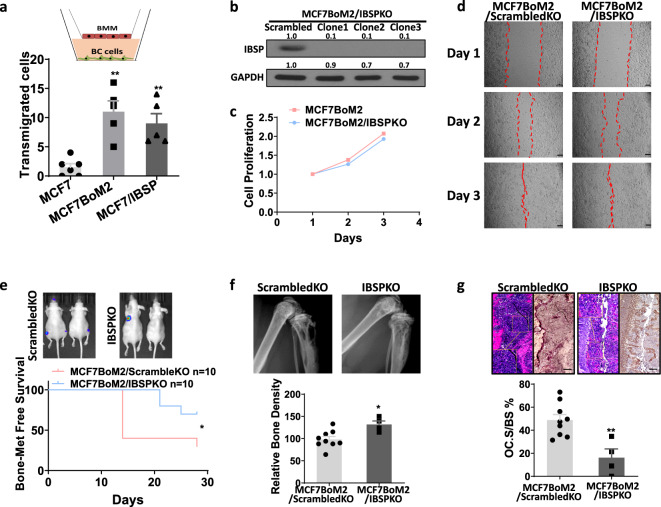
ER^+^ breast cancer cells secrete IBSP to recruit osteoclast precursors. **a** Trans-well migration assays were performed to measure the migration ability of BMMs towards breast cancer cells. BMMs were seeded on the luminal side and breast cancer cells (MCF7, MCF7BoM2, and MCF7/IBSP) were seeded on the abluminal chamber. Data are presented as mean values ± SEM. **b** CRISPR-Cas9 mediated knockout of IBSP in MCF7BoM2. Three knockout clones were confirmed by western blot. The band intensity was quantified and normalized to that of control (left panels). **c** The selected clones were mixed and designated as MCF7BoM2IBSPKO. Cell proliferation was measured for MCF7BoM2 and MCF7BoM2/IBSPKO by MTS assay. A two-sided student’s *t*-test was performed on day 3 and *p* = 0.0496 (*n* = 3 vs 3). Data are presented as mean values ± SEM. **d** Cell migration ability of MCF7BoM2 and MCF7BoM2/IBSPKO was measured by wound-healing assay. Scale bar, 100 µm. **e** MCF7BoM2/ScrambledKO and MCF7BoM2/IBSPKO (500,000/0.1 ml PBS) were transplanted into female nude mice via intracardiac injection. The growth of bone metastasis was monitored by measuring the luciferase activity by IVIS Bioimager, and the Kaplan–Meier analysis was done for bone metastasis-free survival (*p* = 0.0496, HR = 4.101). **f** The tumor-bearing bones were imaged by X-ray, and the relative bone densities were quantified by ImageJ (*p* = 0.0124, *n* = 9 vs 4). Two-sided student’s *t*-tests were performed. Data are presented as mean values ± SEM. **g** OC surface relative to the bone surface was calculated and compared after TRAP staining. H&E staining of the same field was shown together with the TRAP staining (*p* = 0.0036, *n* = 9 vs 4). Two-sided student’s *t*-tests were performed. Data are presented as mean values ± SEM. Scale bar, 100 µm.

Next, we examined the role of IBSP in osteolytic bone metastasis induced by miR-19a. IBSP is known to act as a ligand of integrin αVβ3 receptor that is highly expressed on the surface of OC^40^. A previous study found that IBSP knockout in mice resulted in decreased OC number in bones^40^. Integrin αVβ3 is known to be associated with OC migration and adhesion^50,51^, and IBSP was previously found to be a cell-secreted protein with bone matrix binding ability^52^. Therefore, we hypothesize that IBSP secreted from the bone lodging cancer cells can recruit the OC precursors to the micro-metastatic site followed by creating an OC precursor-enriched niche. Such an environment enhances the uptake of miR-19a-containing exosomes by the OC precursors in a concentration-dependent manner. In a trans-well migration assay, we found that both MCF7BoM2 and MCF7/IBSP recruited more OC precursors to abluminal chambers compared to MCF7 (Fig. 5a), which supports our hypothesis that IBSP attracts OC precursors. We then performed the knockout of IBSP using the CRISPR-Cas9 system in MCF7BoM2. To rule out the clonal effect, three clones that showed no IBSP expression and no alteration of growth ability were selected and mixed into one heterogeneous line, MCF7BoM2/IBSPKO (Fig. 5b, c and Supplementary Fig. 7a). The knockout of IBSP expression did not alter the cell migration ability of MCF7BoM2/IBSPKO (Fig. 5d). However, the bone-metastatic ability was significantly compromised by IBSP knockout in MCF7BoM2/IBSPKO cells in the xenograft mouse model (Fig. 5e). Loss of IBSP also preserved the density of tumor-bearing bones (Fig.5f). Results from TRAP staining of the tumor-bearing tibias also suggested that the IBSP knockout led to decreased osteoclastogenesis (Fig. 5g). These results strongly support our hypothesis that IBSP recruits OC cells to the tumor lesions in the bone, which is essential for the uptake of exosomes and establishment of bone metastasis of ER^+^ breast cancer cells.

### CGA blocks cooperative function of miR-19a and IBSP in ER^+^ BCBM

**Fig. 6:**
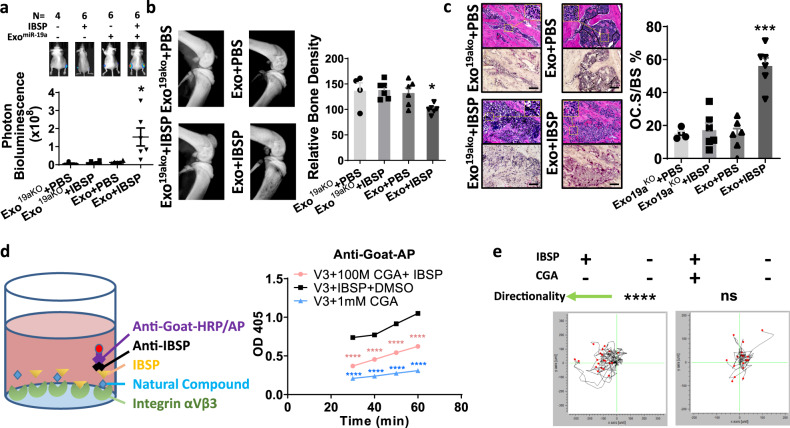
IBSP and miR-19a cooperatively induce osteoclastogenesis and promote cancer cell growth in the bone. **a**, **b** MCF7 cells were injected into the tibias of mice. These mice also received 2 µg exosomes prepared from MCF7BoM2 and 0.5 µg recombinant IBSP injected into the tibias every 2 days. The exosomes prepared from MCF7BoM2/miR-19aKO and PBS were used as controls. After three weeks, the bioluminescence signals of tibial bones were measured. Two-sided student’s t-tests were performed. *p* = 0.4670 (Exo^19aKO^ + PBS vs Exo^19aKO^ + IBSP, *n* = 4 vs 6), *p* = 0.1949 (Exo^19aKO^ + PBS vs Exo + PBS, *n* = 4 vs 6), *p* = 0.0446 (Exo^19aKO^ + PBS vs Exo + IBSP, *n* = 4 vs 6). Data are presented as mean values ± SEM (**a**). The bones were then removed and bone densities were measured by X-ray. Two-sided student’s *t*-tests were performed; *p* = 0.9280 (Exo^19aKO^ + PBS vs Exo^19aKO^ + IBSP, *n* = 4 vs 6), *p* = 0.9143 (Exo^19aKO^ + PBS vs Exo + PBS, *n* = 4 vs 6), *p* = 0.0257 (Exo^19aKO^ + PBS vs Exo + IBSP, *n* = 4 vs 6) Data are presented as mean values ± SEM (**b**). **c** The removed bones were decalcified, sectioned, and examined for the amount of OC surface by TRAP staining followed by quantification using the ImageJ. Two-sided student’s *t*-tests were performed; *p* = 0.7224 (Exo^19aKO^ + PBS vs Exo^19aKO^ + IBSP, *n* = 4 vs 6), *p* = 0.8773 (Exo^19aKO^ + PBS vs Exo + PBS, *n* = 4 vs 6), *p* = 0.0002 (Exo^19aKO^ + PBS vs Exo + IBSP, *n* = 4 vs 6). Data are presented as mean values ± SEM. **d** Integrin αVβ3 was seeded on the bottoms of 96-well plates. IBSP was then added to the well in the presence or absence of the library of natural compounds. The bound IBSP was measured by colorimetric substrates of alkaline phosphatase (AP). The OD 405 was compared between CGA-treated groups and the DMSO-treated group. Two-sided student’s *t*-tests were performed; *p* < 0.0001 (*n* = 3) in all groups. CGA was found to most significantly inhibit the binding of IBSP to integrin αVβ3. Data are presented as mean values ± SEM. **e** The role of IBSP as a chemoattractant of osteoclast cells, and the inhibitory effect of CGA on this chemoattraction were examined by the µ-Slide Chemotaxis assay. The movement of 20 OC cells with the highest mobility was tracked and the directionality was calculated by Chemotaxis and Migration Tool. *p*-value was calculated with the Rayleigh test (left: *p* = 0.0000002, right: *p* = 0.194763).

We hypothesized that cooperation between exosomal miR-19a and IBSP generates the osteolytic bone microenvironment, enabling the ER^+^ tumor growth in the bone. Therefore, we examined the changes in bone microenvironment induced by directly injecting IBSP and exosomal miR-19a prepared from MCF7BoM2 into the tibial bone of mice every two days without tumor cell injection. This treatment resulted in a significant increase in the number of OC at the treatment site (Supplementary Fig. 7b). We also found that the DiD-labeled exosomes from MCF7BoM2 were incorporated into OC cells when they were injected into the tibial bone (Supplementary Fig. 7c). These results suggest that exosomal miR-19a and IBSP cooperatively create an osteolytic bone microenvironment. We then tested whether such change could enhance the growth of primary MCF7 cells in the mice’s tibias (Fig. 6a). MCF7 cells were transplanted into the tibial bone. At the same time, both IBSP and exosomes from MCF7BoM2 were given every two days into the tumor site. To evaluate the roles of exosomal miR-19a, exosomes from MCF7BoM2/miR-19aKO were used as a control. We found that the injection of miR-19a-containing exosomes and IBSP significantly promoted tumor growth (Fig. 6a) in the bones, which resulted in a decrease in bone density (Fig. 6b) and an increase in OC activity (Fig. 6c). These results suggest the indispensable roles of exosomal miR-19a and IBSP in bone metastasis of ER^+^ breast cancer cells. Consistent with these results, knockout of miR-19a or IBSP from MCF7BoM2 significantly decreased the bone-metastatic ability of cancer cells, while ectopic expression of each factor alone did not rescue the effect. (Supplementary Fig. 7d, e).

**Fig. 7:**
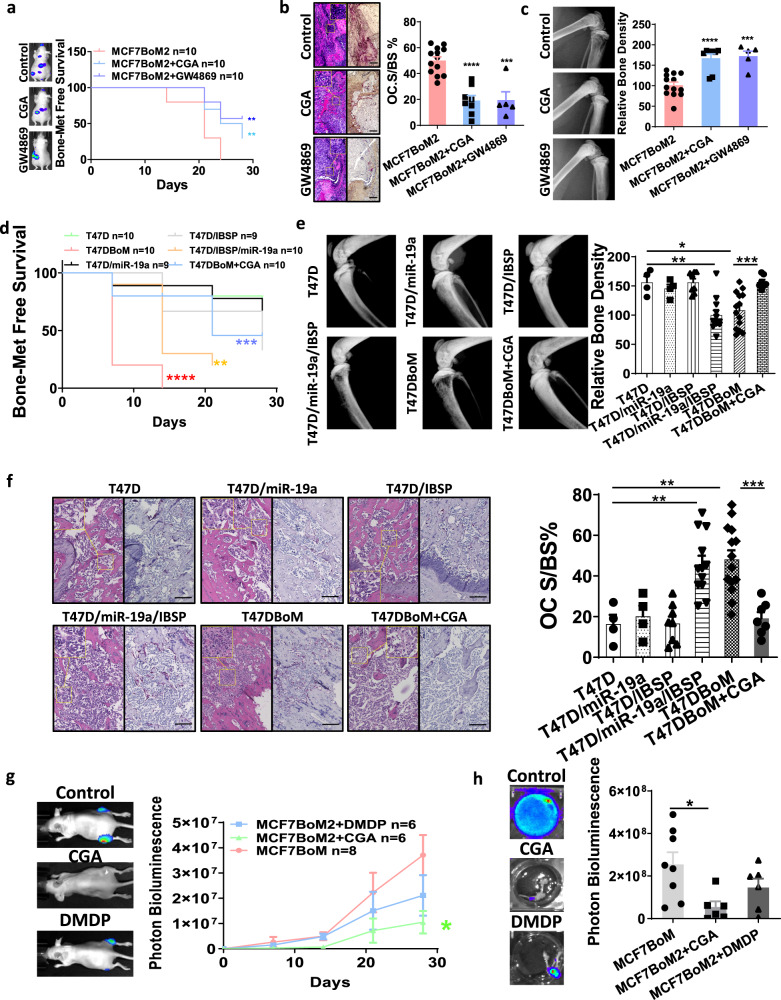
CGA blocks cooperative function of miR-19a and IBSP in ER^+^ BCBM. **a** The mice were treated with 2 µg exosomes prepared from MCF7BoM2 and 0.5 µg recombinant IBSP by injecting them into the tibias every two days. The exosomes from MCF7BoM2/miR-19aKO and PBS were used as controls. After two weeks, the bones were removed, and the TRAP staining of the bones was performed. The OC surface of the bone surface was quantified by ImageJ and compared by the two-sided student’s *t*-test. *p* = 0.0181 (PBS + Exo^19aKO^ vs IBSP + Exo), *p* = 0.2436 (PBS + Exo^19aKO^ vs PBS + Exo), *p* = 0.4594 (PBS + Exo^19aKO^ vs IBSP + Exo^19aKO^). *n* = 5 in all groups. **b** The ratio of OC surface relative to the bone surface was calculated after TRAP staining. *p* < 0.0001 (MCF7BoM2 vs MCF7BoM2 + CGA, *n* = 13 vs 8), *p* = 0.0001 (MCF7BoM2 vs MCF7BoM2 + GW4869, *n* = 13 vs 5). Representative H&E staining of the same field was shown (left panel) together with the TRAP staining (right panel). Scale bar, 100 µm. Two-sided student’s *t*-tests were performed. Data are presented as mean values ± SEM. **c** The legs of tumor-bearing mice were imaged by X-ray, and the bone density was measured by ImageJ. Two-sided student’s *t*-tests were performed. *p* < 0.0001 (MCF7BoM2 vs MCF7BoM2 + CGA, *n* = 13 vs 8), *p* = 0.0002 (MCF7BoM2 vs MCF7BoM2 + GW4869, *n* = 13 vs 5). Data are presented as mean values ± SEM. **d** T47D, T47DBoM, and T47D with ectopic expression of miR-19a or/and IBSP were intra-cardially injected into mice. One group of mice injected with T47DBoM also received CGA treatment. The growth of bone metastasis was monitored by IVIS Bioimager, and the Kaplan–Meier analysis and log-rank tests were performed for bone metastasis-free survival. *p* = 0.000036 (T47D vs T47DBoM); *p* = 0.0078 (T47D vs T47D/IBSP/miR-19a); *p* = 0.0003 (T47DBoM vs T47DBoM + CGA). **e** The legs of tumor-bearing mice were imaged by X-ray, and the bone density was measured by ImageJ. *p* = 0.0027 (T47D vs T47D/IBSP/miR-19a, *n* = 4 vs 12); *p* = 0.0111 (T47D vs T47DBoM, *n* = 4 vs 14); *p* = 0.0009 (T47DBoM vs T47DBoM + CGA, *n* = 14 vs 7). Two-sided student’s *t*-tests were performed. Data are presented as mean values ± SEM. **f** The ratio of OC surface relative to the bone surface was calculated and compared after TRAP staining. H&E staining of the same field was shown (left panels) together with the TRAP staining (right panels). Scale bar, 100 µm. *p* = 0.0028 (T47D vs T47D/IBSP/miR-19a, *n* = 4 vs12); *p* = 0.0023 (T47D vs T47DBoM, *n* = 4 vs 14); *p* = 0.0003 (T47DBoM vs T47DBoM + CGA, *n* = 14 vs 7). Two-sided student’s *t*-tests were performed. Data are presented as mean values ± SEM. **g** One week after intra-tibia injection of MCF7BoM2, mice were treated with CGA or DMDP. The growth of bone tumors was monitored by measuring the luciferase activity by IVIS Bioimager. Two-sided student’s *t*-tests were performed. *p* = 0.0215 (MCF7BoM2 vs MCF7BoM2 + CGA, *n* = 8 vs 6), *p* = 0.1920 (MCF7BoM2 vs MCF7BoM2 + DMDP *n* = 8 vs 6). Data are presented as mean values ± SEM. **h** After 24 days, the legs were removed and the bioluminescence of each leg was quantified. Two-sided student’s *t*-tests were performed. *p* = 0.0144 (MCF7BoM2 vs MCF7BoM2 + CGA, *n* = 8 vs 6), *p* = 0.1747 (MCF7BoM2 vs MCF7BoM2 + DMDP *n* = 8 vs 6). Data are presented as mean values ± SEM.

Because IBSP plays a critical role in recruiting OC precursor cells and enabling the delivery of exosomal miR-19a to promote bone metastasis, targeting IBSP could be an effective preventive strategy to block the IBSP/exosome pathway, and decrease the risks of bone metastasis/recurrence. Therefore, we used a competitive binding assay to screen a natural compounds library aiming at identifying compound(s) that block the binding of IBSP with its receptor integrin αVβ3. In this assay, we layered integrin αVβ3 on the bottom of wells, followed by adding IBSP in the presence or absence of 176 natural compounds. The binding of IBSP protein to integrin αVβ3 was quantified using an enzyme-tagged antibody to measure the inhibitory effect of natural compounds. We found that Chlorogenic acid (CGA) most significantly inhibited the binding of IBSP to integrin αVβ3 (Fig. 6d). To ensure that the inhibitory ability is not a result of direct interaction between the natural compound and the enzyme tag, we repeated our screening with a different enzyme-tagged antibody (Supplementary Fig. 8a). In a chemotaxis assay, we found that CGA significantly blocked the recruitment of OC precursors (mBMM) by IBSP (Fig. 6e). On the other hand, CGA did not directly suppress the growth or migration of cancer cells (Supplementary Fig. 8b, c). These data indicate CGA is a competent inhibitor of the recruitment of OC by IBSP. Importantly, in the xenograft model, both CGA and exosome inhibitor GW4869 effectively suppressed ER^+^ breast cancer bone metastasis (Fig. 7a), while no notable side effects were observed with the mice received CGA treatment (Supplementary Fig. 8d, e). It should be noted that the inhibitory effect of CGA on bone metastasis is not a result of its effect on tumor cell growth, because CGA did not affect the primary tumor growth (Supplementary Fig. 8f, h). On the other hand, CGA did not suppress the bone metastasis of 231BoM cells in our ER^-^ breast cancer xenograft model (Supplementary Fig. 8i). These results demonstrate that CGA is a promising preventive reagent for bone metastasis of ER^+^ breast cancer. With either CGA treatment or exosome blockade, the bone-metastatic lesions showed significantly decreased osteoclastogenesis (Fig. 7b) and increased bone density (Fig. 7c). We also confirm our findings in the T47D xenograft mouse model. When the T47D cells were implanted into mice by intracardiac injection, ectopic expression of miR-19a and IBSP significantly increased the rate of bone metastasis (Fig. 7d), decreased the bone density of the metastatic lesions (Fig. 7e), and increased the OC activities (Fig. 7f). The CGA treatment significantly decreased the rate of bone metastasis, and rescue the osteolytic changes in the bone lesions (Fig. 7d–f). Interestingly, when we generated organ culture of tumor tissues from various distant metastatic lesions in the T47D inoculated mice, we found significantly more exosomal miR-19a in the culture media of bone-metastatic lesions compared to other metastatic sites (Supplementary Fig. 8j). We also examined whether CGA was able to suppress the established bone metastasis. We first transplanted MCF7BoM2 into the tibial bone of mice. After confirming the tumor growth by measuring the bioluminescent signals, CGA or bisphosphonate-DMDP (Dichloromethylenediphosphonic acid disodium salt) was given to the mice. We found that CGA was able to suppress the cancer cell growth of established tumors in the bone more effectively compared to the control group or the bisphosphonate treatment group (Fig. 7g, h).

## Discussion

**Fig. 8:**
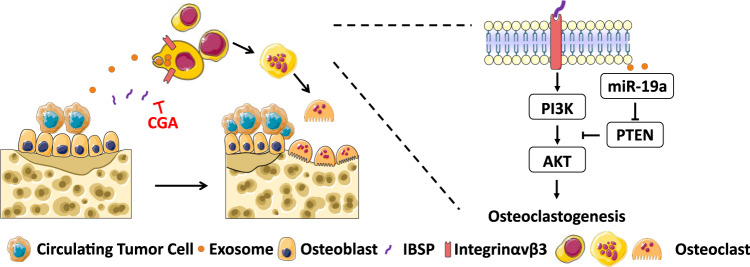
Proposed mechanism of exosomal miR-19a and IBSP mediated bone metastasis in ER^+^ breast cancer. In the early metastatic niche, ER^+^ breast cancer cells secrete IBSP as a chemoattractant to recruit precursors of OC cells, creating an OC precursor-enriched microenvironment. OC precursors incorporate exosomes from breast cancer cells, and miR-19a is transported into OC precursors. Inside the OC precursors, miR-19a suppresses PTEN expression, activates NF-κB and AKT pathways, and promotes osteoclastogenesis. Mature OC cells induce bone resorption, liberating growth factors from the bone matrix, which further promote the growth of cancer cells.

ER^+^ breast cancer patients have a considerably higher risk of bone metastasis^20,22,53–55^ compared to other subtypes. In this study, we identified exosomal miR-19a and IBSP as key secreted factors from ER^+^ bone-metastatic cells. Ectopic expression of one factor alone did not increase bone lesions in our animal models. However, upregulation of both factors together significantly promoted the tumor growth in the bone and osteoclastogenesis, indicating the cooperative roles of exosomal mIR-19a and IBSP in promoting osteolytic bone metastasis. We found that ER^+^ cancer cells secrete IBSP as a chemoattractant for precursors of OC cells, while exosomal miR-19a enhances the osteoclastogenesis in the early metastatic site, which promotes the bone colonization of ER^+^ breast cancer cells (Fig. 8). Importantly, in both serum and transcriptome profiles of breast cancer patients, we verified that miR-19a and IBSP are correlated with bone metastasis and recurrence status. While the serum of advanced breast cancer patients in this study was collected during the course of treatment with tumor burden, we did not see a significant difference in the expression of exosomal miR-19a and IBSP between groups with different treatment histories (Supplementary Data 5). These data suggest the potential use of exosomal miR-19a and IBSP of serum as liquid biomarkers to stratify ER^+^ breast cancer patients with a high risk of bone recurrence.

MiR-19a is located in the miR-17-92 cluster (oncomiR-1), which is characterized as a set of mammalian microRNA oncogenes^56^. The tumor-promoting role of miR-19a has been reported in multiple types of cancers^57–61^, and it is known to directly target tumor suppressor PTEN^42,62,63^. It is also predicted to target ER. However, overexpression of miR-19a did not alter the level of ER in breast cancer cells^64^, and the exact role of miR-19a in breast cancer is yet poorly understood. The previous study has characterized miR-19a targets in breast cancer by quantitative proteomic approach^30^. No growth-suppressive effect was found when those targets were ectopically expressed, suggesting that endogenous miR-19a does not directly alter the growth of cancer cells per se. Consistent with their finding, the results of our study revealed that modulation of miR-19a expression in breast cancer cells did not alter the growth or migration abilities in vitro. Interestingly, ectopic expression of miR-19a did not affect the expression of PTEN in the cancer cells (data not shown). A possible explanation is that miR-19a is actively sorted out of cancer cells by exosomes. Accordingly, miR-19a expression is positively correlated with osteolytic bone metastasis in vivo, strongly suggesting a role of miR-19a as a key modulator of tumor microenvironment in the process of bone metastasis of breast cancer. In the “vicious cycle of bone metastasis”^65^, breast cancer cells are known to interact with host cells to generate a favorable microenvironment for cancer cell growth in the bone through direct cell–cell contact or via secreted proteins^66–68^. We found that the oncogenic role of miR-19a in ER^+^ breast cancer is dependent on its secretory nature as exosomes. Exosomal miR-19a secreted from tumor cells are internalized into the OC precursor and induces the differentiation of OC into mature OC. OC osteoclastogenesis is one of the key steps related to the establishment of bone metastasis, and miR-19a augments this process by activating the p-AKT pathway. Therefore, this study revealed a specific role of miR-19a in ER^+^ breast cancer bone metastasis. The nature of miR-19a encapsulated inside exosomes also makes it a promising candidate as a circulating biomarker for diagnostic and prognostic purposes^69–72^.

IBSP constitutes around 12% of the non-collagenous proteins in human bone and it is synthesized by bone resident cells. Several types of cancers, including colon, breast, prostate, and lung cancers were reported to have upregulated IBSP expression^73^. In breast cancer, IBSP was also detected in cancer cells^74^, and we found that IBSP is only expressed in breast cancer cells but not in normal breast tissue, making it a good candidate as a tumor-specific biomarker. Indeed, IBSP was previously reported to be associated with poor survival of breast cancer patients^75^. Interestingly, it was also found to be positively associated with bone metastasis, but negatively associated with lung metastasis in breast cancer^75^. These observations can be well explained by our results, as we found IBSP regulates bone metastasis in ER^+^ breast cancer. ER^+^ breast cancer is known to have a higher incidence of bone metastasis compared to other subtypes, while they have a lower incidence of lung metastasis^55^. By classifying subtypes of breast cancer patients, we have shown the feasibility of using IBSP, together with exosomal miR-19a to predict the risk of bone metastasis in ER^+^ patients. More importantly, we clarified a cooperative pathway between IBSP and miR-19a, that mobilizes the bone microenvironment to guide the ER^+^ breast cancer cells to the bone and support their cell growth. However, in this study, we did not observe a regulatory effect in gene expression between miR-19a and IBSP (Supplementary Fig. 2a, b). Therefore, the increased bone metastasis is likely to be a result of the colonial selection of high miR-19a/IBSP cells that adapt well to the early metastatic niche due to the interaction with OC cells.

Currently, there is no effective and safe regimen to prevent bone recurrence in breast cancer. The present bone-targeted therapy does not improve the survival of breast cancer patients, and patients with bone metastases still rely on systemic treatment. However, patients with bone recurrence develop resistance to systemic treatment as the cancer cells acquired resistance to these drugs at an early stage. In this study, we found that CGA blocks the interaction between IBSP and OCs, inhibiting the pro-osteoclastogenesis effect of cancer-derived exosomes. CGA is abundant in the extract from plants, and it has minimum side effects for long-term preventive treatment. By using exosomal miR-19a and IBSP to predict the risk of bone metastasis, ER^+^ breast cancer patients could benefit from this agent in preventing late-onset recurrence in the bone. CGA could also be used as a combination therapy to treat ER^+^ breast cancer patients with bone metastasis.

Collectively, our study identified exosomal miR-19a and IBSP as mediators of cell–cell communication between breast cancer cells and OC cells, which promotes the vicious cycle of bone metastasis in ER^+^ breast cancer. Importantly, the natural compound CGA can block this process. This study sheds light on the molecular mechanism of bone metastasis in ER^+^ breast cancer, which warrants further investigation on the miR19a-IBSP axis for the development of an effective therapeutic strategy.

## Methods

### Cells and cell culture

Human breast carcinoma cell lines, MDA-MB-231, MCF7, T47D, and mouse monocyte/macrophage cell line RAW 264.7 were purchased from American Type Tissue Culture Collection (ATCC, Manassas, VA, USA). MCF7BoM2 and 231BoM-1833, the bone-metastatic derivative of breast cancer cell lines were kindly provided by Dr. Joan Massagué (Memorial Sloan-Kettering Cancer Center)^76,35^. To select bone-tropic T47DBoM, 100,000 T47D cells were injected into the left cardiac ventricle of athymic-nu/nu mice. Subsequently, bone-metastatic tumor cells were isolated from the tibias. Three rounds of selection were performed to establish the T47DBoM cell line. Luciferase-labeled cells were generated by infecting the lentivirus carrying the firefly luciferase gene. MDA-MB-231, MCF7 cells and their variants, as well as RAW264.7, were cultured in DMEM medium supplemented with 10% FBS and antibiotics. Murine bone marrow-derived monocytes and macrophages (BMMs) were generated as previously described^77^. Briefly, bone marrow cells were harvested by flushing the femurs and tibias of six to eight-week-old C57BL/6 mice (The Jackson Laboratory, Bar Harbor, ME, USA) with minimum essential medium-α (α-MEM; Invitrogen, Carlsbad, CA, USA). The cells were then washed, centrifuged, and incubated in complete α-MEM containing 10% heat-inactivated fetal bovine serum and penicillin/streptomycin for 18 h to separate the floating and adherent cells. The floating cells were collected and cultured in complete α-MEM supplemented with 50 ng/ml murine recombinant Macrophage Colony Stimulating Factor (M-CSF; Peprotech, Rocky Hill, NJ, USA). After 3 days, non-adherent cells were washed out and the adherent cells were used as BMMs.

### Animal experiments

For experimental metastasis assay, cancer cells in 100 μl of PBS were injected into the left cardiac ventricle of the athymic-nu/nu mice (7–8 weeks). For intratibial implantation of cancer cells, cells with or without ectopic expression of miR-19a and IBSP were injected into the tibia at a final concentration of 100,000 cells/10 μl. To investigate the effect of miR-19a and IBSP on the primary tumor, cancer cells (1 × 10^6^) in 50 µl PBS were mixed with Matrigel matrix (Corning, Corning, NY, USA) at 1:1 ration, and implanted into mammary fat pads of athymic-nu/nu mice. All mice received implantation of 1.7 mg estradiol continuous release pellets (Innovative Research of America). To confirm a successful injection, we immediately monitored photon flux from the whole body of the mice after injection. The growth of tumors in the bones was monitored and quantified by measuring luminescence using IVIS Xenogen bioimager (Caliper Life Science), and the images were analyzed using Living Image (Caliper Life Science) version 4.7.3 software and Aura software (Spectral Instruments Imaging, LLC) version 3.2. For CGA (Sigma-Aldrich, St. Louis, MO, USA) treatment, the compound dissolved in deionized water was administered orally at doses of 200 mg/kg body weight per day. For GW4869 (Cayman Chemical, Ann Arbor, MI, USA) treatment, the drug was intraperitoneally (i.p.) injected at the dose of 2.5 μg/g every three days. For DMDP (Dichloromethylenediphosphonic acid disodium salt) treatment, the drug was intraperitoneally (i.p.) injected at the dose of 10 mg/kg twice a week. For in vivo exosome uptake assay, the mice were treated with 2 µg Vybrant DiD-labeled exosomes with or without 0.5 µg recombinant IBSP by injecting them into the tibias every two days. After two weeks, the bones were extracted and sectioned with a diamond knife. The sectioned bone was then stained with FITC-CTSK (Cathepsin K) and DAPI. The co-localization of OC and exosome was examined by Keyence All-in-one Fluorescence Microscope (BZ-X700). For measurement of the percentage of OC surface per bone surface (OC.S/BS%), bioluminescence and H&E staining were performed to identify the metastatic sites. For each of the mice, all the bone-metastatic sites were TRAP stained and cumulative osteoclast surface and bone surface were measured by ImageJ (FIJI, 1.52n)^78^.

### X-ray imaging

On days 28 before euthanization, X-ray images of the mice were captured with Faxitron Multifocus 10 × 15 Digital Radiography System. Mice were anesthetized and placed dorsal side up in the X-ray chamber with legs in the capture region. X-ray images of the tibias were analyzed using ImageJ (ImageJ 1.8.0_112; 64-bit; National Institute of Health) according to previous publications^79,80^ and relative bone density was quantified. The image was calibrated to a standard aluminum wedge. The average mean grayscale value of the area of interest was recorded after the value of background soft-tissue was subtracted.

### Human L1000 array

The cell culture supernatants were dialyzed and biotinylated according to the manual of L-Series Human Antibody Array 1000 (RayBiotech, Peachtree Corners, GA, USA). The glass slide arrays were then locked and incubated with biotin-labeled samples to allow the binding of target proteins to the pre-printed capture antibodies. Streptavidin-conjugated fluorescent dye is then used to interact with the bound proteins. Finally, when the slide is dried, laser fluorescence scanning was performed with GenePix® 4000B Microarray Scanner to visualize signals. To compare the expression of each protein target, Protein Array Analyzer was used to quantify the signals.

### Gene knockout and overexpression

IBSP and miR-19a knockout was carried out with CRISPR/Cas9 system. lentiCRISPR v2 was a gift from Feng Zhang (Addgene plasmid # 52961; http://n2t.net/addgene:52961; RRID:Addgene_52961) Guide RNAs for IBSP (AGAACTTACTGAGAAAGCAC) and miR-19a (AATCTATGCAAAACTGATGG) were cloned into vectors according to the provided protocol. Lentivirus was prepared by transfection of plasmids into HEK 293T cells. MCF7BoM2 was infected with lentivirus and screened with puromycin. The success of knockout was confirmed by the T7E1 assay (NEB). Single-cell clones were picked by limit dilution. Four miR-19a knockout clones and three IBSP knockout clones were selected. The knockout clones of each gene were mixed to generate the heterogeneous knockout cell lines and they were used for functional assays. The stabilities of the knockout were verified every five passages by PCR and western blot analysis. The plasmid for overexpression of IBSP (LV185867) was purchased from ABM (Applied Biological Materials, Richmond, BC, Canada) and the plasmid for overexpression of miR-19a (PCDH-miR-19a) was a kind gift from Dr. Yin-yuan Mo.

### Osteoclastogenesis assay and tartrate-resistant acid phosphatase (TRAP) staining

Murine BMMs at a density of 1 × 10^6^ cells/ml or RAW264.7 cells at a density of 1 × 10^5^ cells/ml were plated in a chamber slide (Thermofisher, Waltham, MA, USA) and cultured in complete α-MEM supplemented with 50 ng/ml murine recombinant M-CSF, 25 ng/ml murine recombinant RANKL (Peprotech, Rocky Hill, NJ, USA) in the presence or absence of 20 μg/ml exosomes, a concentration used previously in multiple studies^27,66,81,82^. On day 4, TRAP staining was performed using a leukocyte acid phosphatase kit (Sigma-Aldrich, St. Louis, MO, USA) according to the manufacturer’s instructions. The multinucleated TRAP-positive cells from five biological replicates were considered mature OC cells and monitored by Zeiss Axioplan2 microscope. The size of OC cells was measured by ImageJ as reported^83^.

### Bone-resorption assay

To determine the role of exosomal miR-19a in the bone resorption activity of OC cells, pit assay was performed on bone slices (Immunodiagnostic Systems). 50,000 bone marrow-derived OC precursor BMMs were seeded on sterilized bone slices placed in a 96-well plate. The BMMs were cultured with complete α-MEM supplemented with 50 ng/ml murine recombinant M-CSF, 25 ng/ml murine recombinant RANKL (Peprotech, Rocky Hill, NJ, USA) in the presence or absence of 20 μg/ml exosomes. The media was changed every 2–3 days. After two weeks of cell culture, cells were washed, and resorption pits were stained with 1% toluidine blue. The resorption area was photographed under microscopy and the image was analyzed by ImageJ.

### Extracellular vesicles purification and tracking analysis

Extracellular vesicles (EVs) from cells were isolated by ultracentrifugation. Cells were grown in EVs-depleted media for 48 h before CM was collected for EVs purification. To isolate total EVs, CM was centrifuged at 300 × *g* for 10 min to remove cells. EVs were collected by ultracentrifugation at 120,000 × *g* for 70 min. Apoptotic bodies (ABs), microvesicles (MVs), and exosomes were isolated by differential centrifugation as published before^84,85^. Briefly, the harvested CM was centrifuged at 300 × *g* for 10 min to remove cells. ABs were collected by centrifugation at 2000 × *g* for 20 min. The supernatant was centrifuged at 16,500 × *g* for 20 min to collect MVs. After that, the Supernatant was passed through a 0.2 µm filter (Sarstedt, Nümbrecht, GERMANY) to remove the particles larger than 200 nm. Exosomes were then pelleted by ultracentrifugation at 120,000 × *g* for 70 min. The isolated EVs were analyzed and quantified by nanoparticle tracking analysis (NTA) and electron microscope with negative staining.

### Imaging of exosomes by transmission electron microscopy

For negative staining, Purified exosomes were incubated onto discharged 200 mesh copper EM grids and fixed with 2% Paraformaldehyde (PFA). 1% uranyl acetate (UA) solution was used to stain the exosomes on the surface of the EM grids. The exosomes were imaged by FEI Tecnai BioTwin Transmission Electron Microscope. For immunostaining of IBSP and CD63 on the surface of exosomes, Anti-CD63 (1:100) (Santa Cruz Biotechnology, Dallas, Texas, USA) and anti-IBSP (1:100) (Thermofisher, Waltham, MA, USA) were used to incubate the fixed exosomes on the grids. The 10 nm colloidal gold-labeled protein A (1:50)(BOSTER BIOLOGICAL TECHNOLOGY, Pleasanton, CA, USA) was then used to label the primary antibodies. After that, negative staining was performed with 2% uranyl acetate and the grid was imaged by transmission electron microscopy.

### Exosome uptaking assay

Breast cancer cell lines were labeled with lentivirus expressing PalmGFP (a kind gift from Dr. Xandra O. Breakefield). Cells with intense fluorescence were selected by BD FACSAria Cell Sorter. Exosomes labeled with PalmGFP were isolated by differential ultracentrifuge. To monitor the uptake of exosomes in OC cells, RAW264.7 cells were seeded in chamber slides overnight and 20 μg/ml exosomes were added into the media. After 24 h of incubation, the cells were washed and fixed. Cell nuclei were stained with DAPI followed by sealing the slides with coverslips. The uptake of exosomes was confirmed by examining the GFP signal under Olympus FV1200 SPECTRAL laser scanning confocal microscope.

### ExoELISA

To compare the exosome production from cells after ectopic expression of miR-19a and IBSP, exosome concentration in the cell culture media was determined using ExoElisa from System Biosciences (SBI, Mountain View, CA, USA). According to the manufacturer’s instruction, exosomes from a comparable amount of different cell lines were purified by ExoQuick-TC precipitation solution (SBI, Mountain View, CA, USA). Diluted exosomes, together with protein standards, were incubated with a provided microtiter plate. The detection antibody was added to the wells, followed by a horseradish peroxidase enzyme-linked secondary antibody. Finally, TMB ELISA substrate was added to develop the signal, and a stop buffer was used to stop the reaction. OD value was recorded with a spectrophotometric plate reader at 450 nm. The data were normalized to the cell count.

### Immunohistochemistry

Immunohistochemical analysis was carried out for paraffin-embedded, surgically resected specimens of breast cancer bone metastases. Briefly, the sections were de-paraffinized, rehydrated, and heated at 100 °C for 20 min in Tris-buffered saline (pH 9.0) for antigen exposure. They were treated with 3% H_2_O_2_ to block endogenous peroxidase activity, followed by treatment with 5% BSA solution for 30 min. The slides were then incubated with primary antibodies targeting IBSP (Invitrogen, PA5-50633, 1:100) and E-cadherin (Cell Signaling Technology, clone 24E10, #3195, 1:200) for 16 h at 4 °C. After washing in PBS/0.1% Tween-20, the sections were treated with the secondary antibody anti-rabbit (Bio-Rad, #1706515, 1:500). The sections were washed extensively, and DAB substrate chromogen solution was applied followed by counterstaining with hematoxylin. For negative control, we added the rabbit IgG isotype control (Invitrogen, 02-6102, 1:250) instead of primary antibodies during the primary antibody incubation. The expression of the protein of interest was evaluated by H scoring as the sum of the frequency and the intensity (0: none; 1: weak; 2: moderate; 3: strong). The final score = 1 × (% of 1 + cells) + 2 × (% of 2 + cells) + 3 × (% of 3 + cells). IHC scores were determined by concordance among the scores of two independent reviewers.

### MTS assay

Viability of cell lines was quantified by using the CellTiter 96 Aqueous One solution cell proliferation assay kit (Promega, Madison, WI, USA). Cells were plated in 96-well tissue culture plates at a density of 5000 cells per well. At the time of measurement, 20 µl of CellTiter 96 AQueous One solution reagent was added into each well of the 96-well assay plate containing the samples in 100 µl of culture medium. The plate was incubated at 37 °C for 1 h in a humidified, 5% CO_2_ atmosphere. The absorbance at 490 nm was measured and recorded.

### Wound-healing migration assay

The cell migration was estimated by a wound-healing migration assay and monitored by the Olympus IX-70 microscope. Cells were seeded in 12 well cell culture dish and a scratch was made on the monolayer using a sterile tip when cells reach 100% confluence. The distance of migration by cancer cells was measured every 24 h.

### Real-time PCR and western blotting

Total RNA was isolated by using Direct-zol RNA MiniPrep Plus (Zymo Research Corp, Irvine, CA, USA) and miRNeasy Mini Kit (Qiagen, Germantown, MD, USA) for mRNA and microRNA, respectively. We used iScript Reverse Transcription Supermix (Life Science Research, Hercules, CA, USA) and TaqMan MicroRNA Reverse Transcription kit (ThermoFisher Scientific, Waltham, MA, USA) for mRNA and miRNA reverse transcription, respectively. Quantitative PCR (qPCR) for miRNA was conducted by using TaqMan Universal Master Mix II and TaqMan microRNA assays (ThermoFisher Scientific, Waltham, MA, USA). All miRNA assays were purchased from ThermoFisher Scientific. U6 snRNA and RNU48 TaqMan probes were used as endogenous controls for human and mouse cells, respectively. For coding genes, qPCR was conducted by using iTaq Universal SYBR Green Supermix (Life Science Research, Hercules, CA, USA). Primers used in this study were listed in Supplementary Data 1. Western blotting was carried out using general methods. Briefly, the protein was extracted from the cells using RIPA buffer, quantified by Protein Assay (Bio-Rad, Hercules, California, USA), resolved by 10% SDS-polyacrylamide gel, and then electrotransferred to nitrocellulose membrane. Primary antibodies against IBSP (Invitrogen, PA5-50633, 1:500), Phospho-NF-κB p65 (Ser536) (Cell Signaling Technology, clone 93H1, #3033, 1:1000), NF-κB p65 (Cell Signaling Technology, #8242, 1:1000), Phospho-Akt (Ser473) (Cell Signaling Technology, clone D9E, #4060, 1:3000), Akt (Cell Signaling Technology, #9272, 1:2000), HSP70 (Invitrogen, Clone 5A5, #MA3-007, 1:1000), PTEN (Santa Cruz Biotechnology, clone A2B1, sc-7974, 1:1000), CD63 (Santa Cruz Biotechnology, clone MX-49.129.5, sc-5275, 1:500), TSG101 (Santa Cruz Biotechnology, clone C-2, sc-7964, 1:1000), GRP94 (Santa Cruz Biotechnology, clone H-10, sc-393402, 1:500), α-actinin-4 (Santa Cruz Biotechnology, clone G-4, sc-390205, 1:500), Calregulin (Santa Cruz Biotechnology, clone A-9, sc-166837, 1:500), GAPDH (Cell Signaling Technology, clone D16H11, #5174, 1:15,000), α-tubulin (Cell Signaling Technology, #2144, 1:2000) were used. HRP-conjugated anti-mouse IgG (Cell Signaling Technology, #7076) or anti-rabbit IgG (Bio-Rad, #1706515) and mouse IgG kappa binding protein m-IgGκ BP-HRP (Santa Cruz Biotechnology, sc-516102) were used as secondary antibodies. Immunoblotting images were captured by Amersham Imager 600 or the X-ray film processor. Images were quantified with ImageJ software.

### Receptor–ligand in vitro binding assay

The receptor–ligand in vitro binding assay was developed according to previous publications^86,87^. Briefly, a high-binding 96-well plate was coated with integrin αVβ3 in the carbonate-bicarbonate buffer. The plate was sealed and incubated at 4 °C overnight. The plate was then washed and blocked with PBS (0.1% Tween and 1% BSA). IBSP with or without the natural compound library was incubated with coated integrin αVβ3 at room temperature for 2 h. The plate was then washed and further incubated with primary antibody Goat anti-IBSP (R&D SYSTEMS, AF4014, 1:100) or Goat IgG Control (R&D SYSTEMS, AB-108-C, 1:100). Anti-goat IgG tagged with either HRP (horseradish peroxidase) (Invitrogen, A15999, 1:5000) or AP (alkaline phosphatase) (Invitrogen, A16002, 1:2500) was used as the secondary antibody. After washing the plates, the substrate was added, and the plates were incubated for 30 min followed by adding the stop solution. The binding of integrin αVβ3 and IBSP was quantified by measuring the absorbance value with a plate reader.

### μ-Slide chemotaxis assay

The μ-Slide chemotaxis assay was carried out according to the manufacturer’s instructions. Briefly, OC precursors BMMs at 3 × 10^6^ cells/ml were mixed with collagen type I gels and other ingredients before being seeded into the middle channel. Left and right chambers were filled with FBS media with or without IBSP. The movement of cells was tracked according to a previous publication^88^. Time-lapse video microscopy was performed using an inverted Nikon microscope. Cell tracking was performed using the ImageJ software (National Institutes of Health, Bethesda, USA) plugin “Manual Tracking” (Fabrice Cordelières, Institut Curie, Orsay, France), then analyzed with Chemotaxis and Migration Tool (Version 2.0, IBIDI).

### Clinical samples

Clinical samples from breast cancer patients and healthy individuals were obtained from Tumor Tissue and Pathology Shared Resource of Wake Forest Baptist Comprehensive Cancer Center and Cooperative Human Tissue Network. The serum samples from early-stage breast cancer patients were collected before surgery and the serum samples from advanced breast cancer patients were collected during the course of treatment. Population characteristics including patient age, race, diagnosis, metastasis, receptor status, and treatment history were retrieved from medical records, and summarized in Supplementary Data 2 and 3. The patient information of tumor tissue and healthy bone tissue was summarized in Supplementary Data 4.

### MiRNA array, bioinformatics, statistical tests, and reproducibility

EVs from MCF7 and MCF7BoM2 were collected and miRNA was extracted using miRNeasy Micro Kit (Qiagen, Hilden, North Rhine-Westphalia, Germany). Expression profiling of miRNA was performed by using the Affymetrix GeneChip miRNA Array. For calculation of FDR and fold change in differential expression analyses, the ComparativeMarkerSelection module from GenePattern^89^ (Broad Institute, Cambridge, MA, USA) was used. For clustering and visualization, the HierarchicalClustering and HierarchicalClusteringViewer modules from GenePattern were used. For metastasis-free survivals, Kaplan–Meier curves were plotted using the time to event as the outcome. Statistical differences in survival across groups were assessed using the log-rank test. For other analyses, student’s *t*-tests (two-sided) were used unless otherwise noted. All other statistical analyses were performed using the GraphPad Prism 8 Program (GraphPad Software, San Diego, CA, USA). Asterisk: *p < 0.05, **p < 0.01, ***p < 0.001, ****p < 0.001, ^ns^*p* ≥ 0.05. Representative experiments were repeated independently three times with similar results.

### Reporting summary

Further information on research design is available in the Nature Research Reporting Summary linked to this article.

## Supplementary information

Supplementary Information

Description of Additional Supplementary Files

Supplementary Data 1

Supplementary Data 2

Supplementary Data 3

Supplementary Data 4

Supplementary Data 5

Reporting Summary

## Data Availability

CEL and expression files associated with the GeneChip miRNA array in this study are deposited at GEO (https://www.ncbi.nlm.nih.gov/geo/) with the accession number GSE176105. The GEO data set (GSE41922, GSE22220, GSE56493, GSE2034), TCGA referenced during the study are available in a public repository from https://www.ncbi.nlm.nih.gov/geo/ and https://portal.gdc.cancer.gov/ websites. The source data underlying Figs. 1–7 and Supplementary Figs. 1–8 are provided as a Source Data file. All the other data supporting the findings of this study are available within the article and its supplementary information files and from the corresponding author upon reasonable request. A reporting summary for this article is available as a Supplementary Information file. Source data are provided with this paper.

## Source data

Source data
