# Supplementary material for: Exosomal miR-19a and IBSP cooperate to induce osteolytic bone metastasis of estrogen receptor-positive breast cancer
Source: Nat Commun. 2021 Aug 31;12:5196. doi: 10.1038/s41467-021-25473-y (PMC8408156; doi:10.1038/s41467-021-25473-y)
Supplement: Supplementary file 1 — Supplementary Information [file 41467_2021_25473_MOESM1_ESM.pdf]

Supplementary information

Exosomal miR-19a and IBSP cooperate to induce osteolytic bone metastasis of estrogen receptor-positive breast cancer

Kerui Wu[1], Jiamei Feng[1,2], Feng Lyu[1,3], Fei Xing[1], Sambad Sharma[1], Yin Liu[1], Shih-Ying Wu[1], Dan Zhao[1], Abhishek Tyagi[1], Ravindra Pramod Deshpande[1], Xinhong Pei[1,4], Marco Gabril Ruiz[1], Hiroyuki Takahashi[5], Shunsuke Tsuzuki[5], Takahiro Kimura[5], Yin-yuan Mo[6], Yusuke Shiozawa[1], Ravi Singh[1] and Kounosuke Watabe[1]*

[1]Department of Cancer Biology, Wake Forest University School of Medicine, Winston-Salem, NC 27157, USA
[2]Mammary Department, Shuguang Hospital Affiliated to Shanghai University of Traditional Chinese Medicine, Shanghai, 201021, China
[3]Department of Breast Surgery, Henan Provincial People's Hospital; People's Hospital OF Zhengzhou University; People's Hospital of Henan University, Zhengzhou, Henan, 450003, China
[4]Department of Breast Surgery, The First Affiliated Hospital of Zhengzhou University, Zhengzhou, Henan 450052, China
[5]Department of Pathology, Jikei University School of Medicine, Minato City, Tokyo 105-8461, Japan
[6]Cancer Institute, University of Mississippi Medical Center, Jackson, MS 39216, USA
* To whom correspondence should be addressed: Kounosuke Watabe Ph.D. Department of Cancer Biology, Wake Forest University School of Medicine, Winston Salem, NC 27157; Phone: 336-716-0231; Fax: 336-716-0255; Email: kwatabe@wakehealth.edu

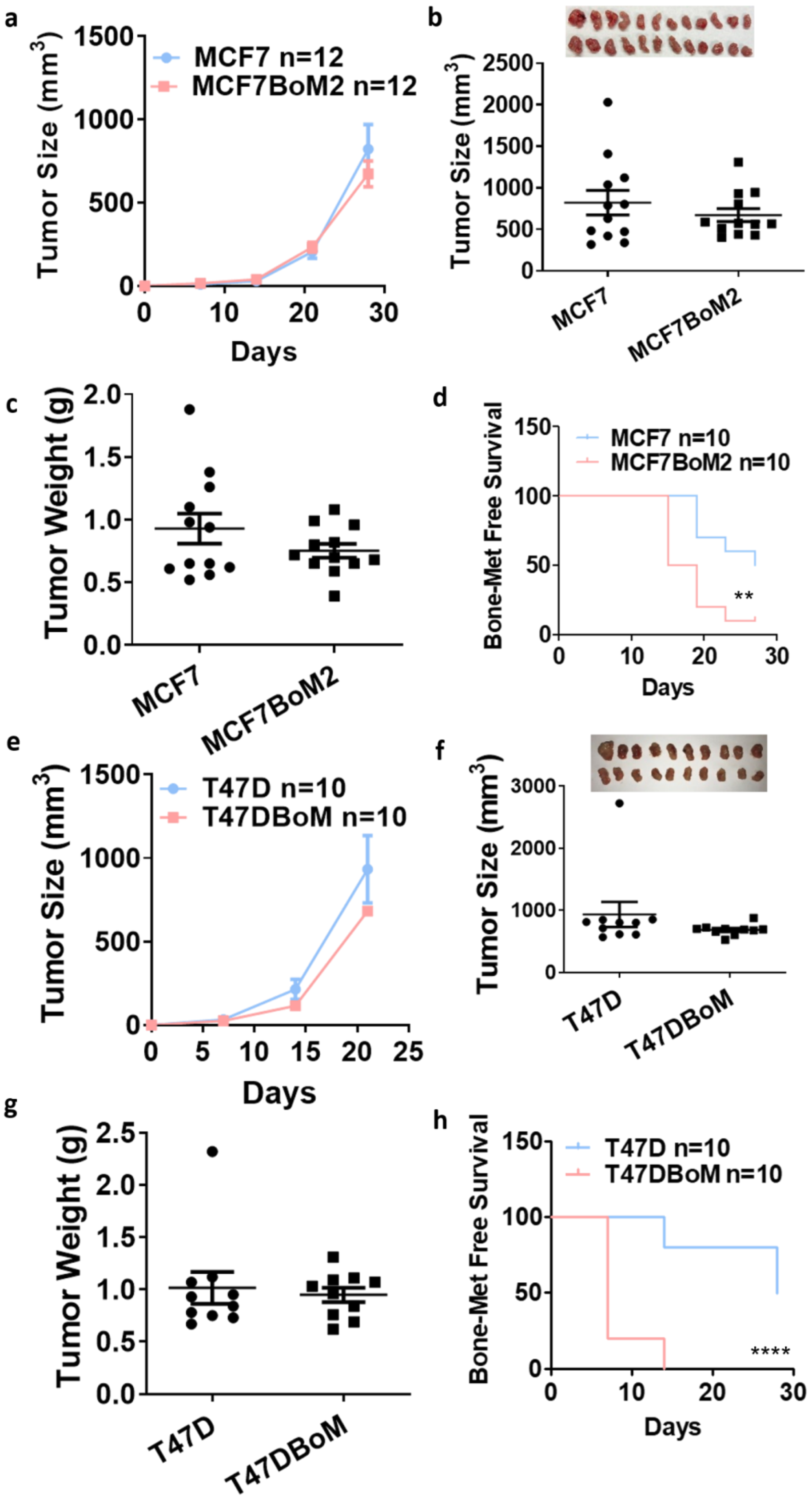

a
Tumor Size (mm3)
1500
1000
500
0
MCF7 n=12
MCF7BoM2 n=12
0
10
20
30
Days
b
Tumor Size (mm3)
2500
2000
1500
1000
500
0
MCF7
MCF7BoM2
c
Tumor Weight (g)
2.0
1.5
1.0
0.5
0.0
MCF7
MCF7BoM2
d
Bone-Met Free Survival
150
100
50
0
MCF7 n=10
MCF7BoM2 n=10
**
0
10
20
30
Days
e
Tumor Size (mm3)
1500
1000
500
0
T47D n=10
T47DBoM n=10
0
5
10
15
20
25
Days
f
Tumor Size (mm3)
3000
2000
1000
0
T47D
T47DBoM
g
Tumor Weight (g)
2.5
2.0
1.5
1.0
0.5
0.0
T47D
T47DBoM
h
Bone-Met Free Survival
150
100
50
0
T47D n=10
T47DBoM n=10
****
0
10
20
30
Days

Supplementary Figure 1. The ER+ bone-tropic breast cancer cells have increased bone metastasis ability

(a) MCF7 and MCF7BoM2 cells were injected into the mammary fat pad of mice. The tumor growth was monitored and compared by measuring the size of tumors. After three weeks, the tumors were removed and their sizes (p=0.3592, n=12) **(b)** and weights (p=0.1921, n=12) **(c)** were measured. Two-sided student's t-tests were performed. Data are presented as mean values +/- SEM. **(d)** MCF7 and MCF7BoM2 cells were intracardially injected into the mice. The growth of bone metastasis was monitored by IVIS Bioimager, and the Kaplan-Meier analysis and log-rank test were performed for bone-metastasis free survival (p=0.0094). **(e)** T47D and T47DBoM cells were injected into the mammary fat pad of mice. The tumor growth was monitored and compared by measuring the size of the tumors. After three weeks, the tumors were removed and their sizes (p=0.2372, n=10) **(f)** and weights (p=0.6884, n=10) **(g)** were measured. Two-sided student's t-tests were performed. Data are presented as mean values +/- SEM. **(h)** T47D and T47DBoM cells were intracardially injected into the mice. The growth of bone metastasis was monitored by IVIS Bioimager, and the Kaplan-Meier analysis and log-rank test were performed for bone-metastasis free survival (p=0.000036).

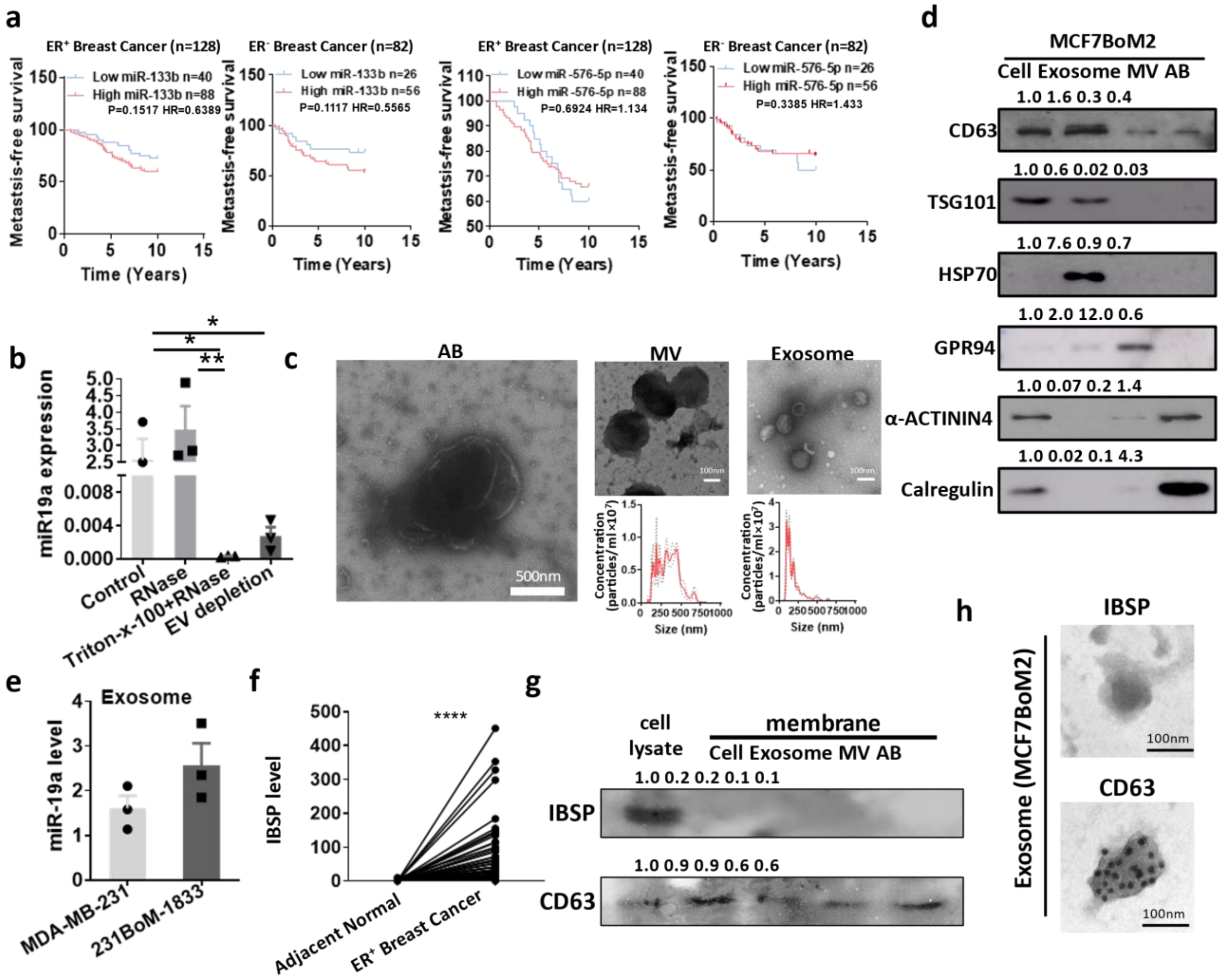

Supplementary Figure 2. **Bone metastatic ER+ breast cancer cells secrete exosomal miR-19a and IBSP**

(a) The Kaplan-Meier analyses for miR-133b and miR-576-5p were performed among breast cancer patients using the GEO dataset (GSE22220). In ER^+ breast cancer cohort, there are 40 low miR-133b/miR-576-5p and 88 high miR-133b/miR-576-5p patients. In ER^- breast cancer cohort there are 26 low miR-133b/miR-576-5p and 56 high miR-133b/miR-576-5p patients. **(b)** Taqman PCR analysis was performed for miR-19a expression in CM from MCF7BoM2 with or without RNase treatment (n=3). Cel-miR-39 was added after RNase treatment/inactivation, and used as the reference gene. Two-sided student's t-tests were performed. P=0.0160 (Control vs Triton-x-100+RNase), p=0.0078 (RNase vs Triton-x-100+RNase), p=0.0160 (Control vs EV depletion). Data are presented as mean values +/- SEM. **(c)** Electron microscopy and NTA analyses for the size distribution of EVs. The results of NTA analyses indicate the average size of MV is 300.3nm and the average size of exosome is 149.6nm. Scale bar, 500nm (AB) and 100nm (MV/Exosome). **(d)** The expression of exosomal markers (CD63, HSP70 and TSG101), MV marker (GPR94) and AB marker (α-Actinin4 and Calregulin) in 10µg lysate of MCF7BoM2 cell and EVs were examined by western blot. **(e)** Taqman PCR was performed for miR-19a expression in exosomes from MDA-MB-231 and 231BoM-1833. P=0.1643 (n=3, two-sided student's t-test). Data are presented as mean values +/- SEM. **(f)** IBSP expression was examined for 70 paired normal breast and ER^+ breast cancer tissues from TCGA (p+0.00000053, two-sided student's t-test). Each line indicates a pair of samples from one patient. **(g)** IBSP expression in cell lysate and membrane fractions from the cell, exosome, MV and AB of MCF7BoM2 was measured by Western Blot. **(h)** Immunostaining of IBSP and CD63 was performed for exosomes prepared from MCF7BoM2. Electron microscopy was performed for the stained exosomes.

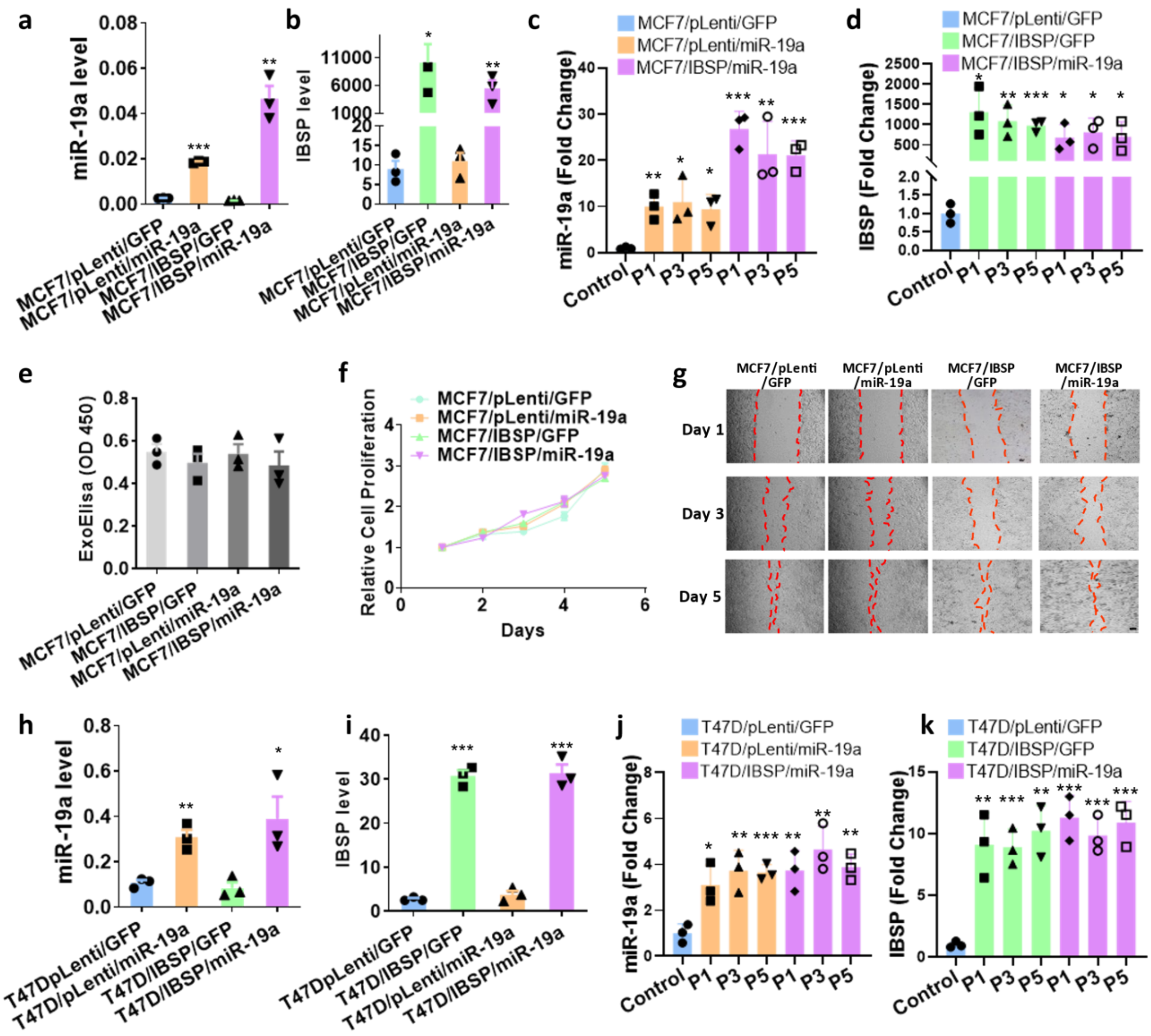

Supplementary Figure 3. Overexpression of IBSP and miR-19a in ER⁺ breast cancer

(a) Taqman PCR was performed for miR-19a expression in MCF7 with miR-19a and/or IBSP ectopic expression. Two-sided student's t-tests were performed. P<0.0001(MCF7/pLenti/GFP vs MCF7/pLenti/miR-19a, n=3), p=0.0013 (MCF7/IBSP/GFP vs MCF7/IBSP/miR-19a, n=3). Data are presented as mean values +/- SEM. **(b)** IBSP expression was measured by PCR in MCF7 with ectopic expression of miR-19a and/or IBSP. Two-sided student's t-tests were performed. P=0.0390(MCF7/pLenti/GFP vs MCF7/IBSP/GFP, n=3), p=0.0213 (MCF7/pLenti/miR-19a vs MCF7/IBSP/miR-19a, n=3). Data are presented as mean values +/- SEM. **(c)** Taqman PCR was performed for miR-19a expression in passages 1, 3 and 5 of miR-19a over-expressed cell lines. Two-sided student's t-tests were performed. P=0.0051, 0.0269, 0.0107, 0.0003, 0.0076, 0.0004 (left to right) and n=3 in each group. Data are presented as mean values +/- SEM. **(d)** IBSP expression was measured by PCR in passages 1, 3 and 5 of IBSP over-expressed cell lines. Two-sided student's t-tests were performed. P=0.019, 0.0094, 0.0003, 0.0245, 0.0169, 0.0293 (left to right) and n=3 in each group. Data are

presented as mean values +/- SEM. **(e)** ExoElisa was performed for the conditioned media from MCF7 with miR-19a and/or IBSP ectopic expression. The OD 450 was recorded and compared. P>0.05 for all two-sided student's t-tests performed between each two of all. N=3 for each group. Data are presented as mean values +/- SEM. **(f)** MTS assay was performed for MCF7 with ectopic expression of miR-19a and/or IBSP. Two-sided student's t tested was performed on day 5 and p>0.05 in all tests performed between any two groups. N=3 in all groups. Data are presented as mean values +/- SEM. **(g)** Wound healing assay was performed for MCF7 with ectopic expression of miR-19a and/or IBSP. Scale bar, 100µm. **(h)** Taqman PCR was performed to measure miR-19a expression in T47D with ectopic expression of miR-19a and/or IBSP. Two-sided student's t-tests were performed. P=0.0047 (T47D/pLenti/GFP vs T47D/pLenti/miR-19a, n=3), p=0.0396 (T47D/IBSP/GFP vs T47D/IBSP/miR-19a, n=3). Data are presented as mean values +/- SEM. **(i)** IBSP expression was measured by PCR in T47D with ectopic expression of miR-19a and/or IBSP. Two-sided student's t-tests were performed. P<0.0001 (T47D/pLenti/GFP vs MCF7/IBSP/GFP, n=3), p=0.0002 (T47D/pLenti/miR-19a vs T47D/IBSP/miR-19a, n=3). Data are presented as mean values +/- SEM. **(j)** Taqman PCR was performed for miR-19a expression in passages 1, 3 and 5 of miR-19a over-expressed cell lines. Two-sided student's t-tests were performed. P=0.0189, 0.0081, 0.0010, 0.0079, 0.0046, 0.0020 (left to right) and n=3 in each group. Data are presented as mean values +/- SEM. **(k)** IBSP expression was measured by PCR in passages 1, 3 and 5 of IBSP over-expressed cell lines. Two-sided student's t-tests were performed. P=0.0055, 0.0008, 0.0015, 0.0006, 0.0006, 0.0006 (left to right) and n=3 in each group. Data are presented as mean values +/- SEM.

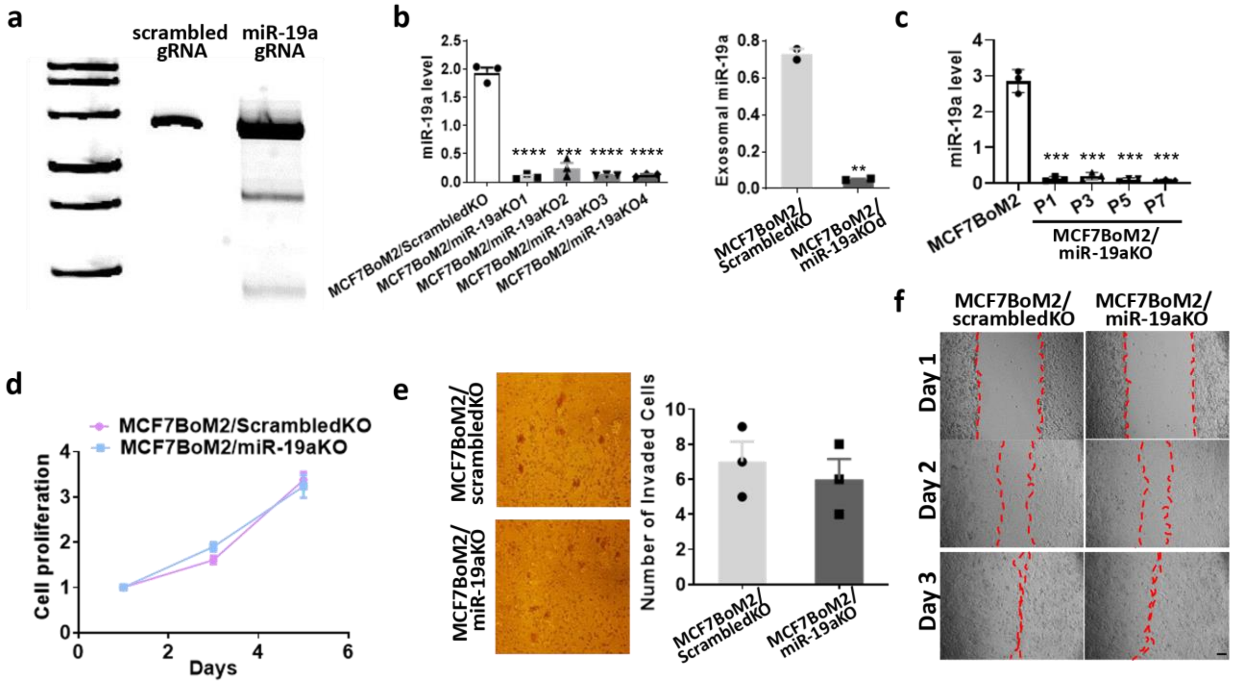

Supplementary Figure 4. Knockout of miR-19a in MCF7BoM2 by CRISPR/Cas9

(a) T7E1 assay was performed for testing gRNA efficiency targeting the stem-loop of miR-19a. The lower bands indicate the cleavage of PCR product by T7E1 that recognizes the mismatch resulted from CRISPR/Cas9-gRNA induced double-strand DNA break. **(b)** Knockout of miR-19a in four clones of

MCF7BoM2/miR-19aKO was confirmed by Taqman PCR. Both endogenous and exosomal levels of miR-19a were measured. MCF7BoM2/miR-19aKO is the mixture of four knockout clones. Two-sided student's t-tests were performed. P=0.000043 (MCF7BoM2/ScrambledKO vs MCF7BoM2/miR-19aKO1); p=0.0002 (MCF7BoM2/ScrambledKO vs MCF7BoM2/miR-19aKO2); p=0.000039(MCF7BoM2/ScrambledKO vs MCF7BoM2/miR-19aKO3); p=0.000041 (MCF7BoM2/ScrambledKO vs MCF7BoM2/miR-19aKO4); p=0.0019 (MCF7BoM2/ScrambledKO vs MCF7BoM2/miR-19aKO). N=3 in all groups. Data are presented as mean values +/- SEM. **(c)** The expression of miR-19a in four different passages of MCF7BoM2/miR-19aKO was examined by Taqman PCR to confirm the stability of the knockout. Two-sided student's t-tests were performed. P=0.0001, 0.0002, 0.0001, 0.0001 (from left to right) and n=3 in each group. MTS assay was performed for MCF7BoM2/ScrambledKO and MCF7BoM2/miR-19aKO. Data are presented as mean values +/- SEM. **(d)** MTS assay was performed for MCF7BoM2 with or w/o miR-19a knockout. Two-sided student's t-test was performed on day 5 and p=0.6282 (n=3 in both groups). Data are presented as mean values +/- SEM. **(e)** Invasion assay was performed for MCF7BoM2/ScrambledKO and MCF7BoM2/miR-19aKO. The effect of miR-19a on cell migration was assessed by the cell number in the abluminal chamber. Two-sided student's t-test was performed (p=0.5734, n=3). Data are presented as mean values +/- SEM. **(f)** Wound healing assay for MCF7BoM2/ScrambledKO and MCF7BoM2/miR-19aKO. The effect of miR-19a on cell migration was assessed by examining the recovery from the scratch. Scale bar, 100µm.

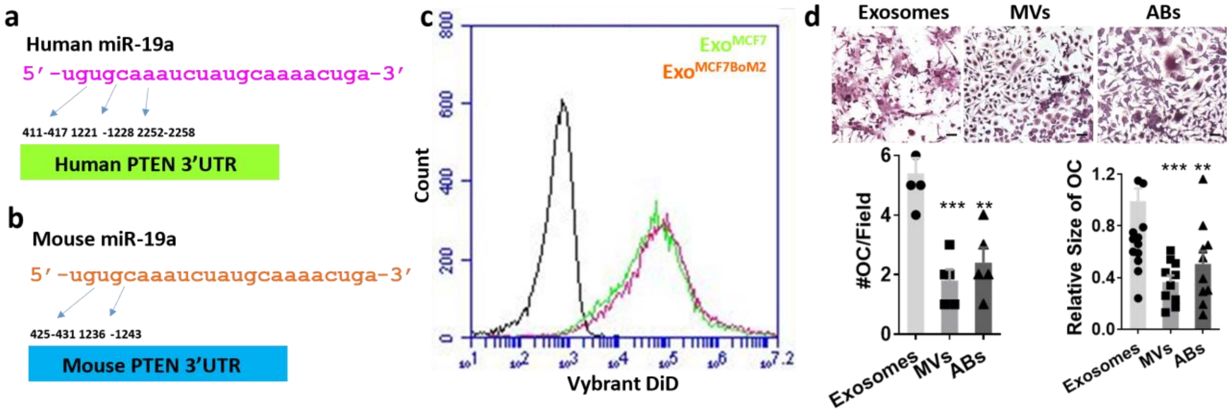

Supplementary Figure 5. miR-19a is conserved between human and mouse and exosomal miR-19a induces OC differentiation

(a) Human miR-19a and its predicted binding sites to 3'UTR of human PTEN. **(b)** Mouse miR-19a and its predicted binding sites to 3'UTR of mouse PTEN. **(c)** Vybrant DiD-labelled exosomes prepared from MCF7 and MCF7BoM2 were used to treat RAW264.7 at 20µg/ml. After 24 hours, the uptake of the exosomes was measured by flow cytometry. **(d)** RAW264.7 was treated with 20µg/ml of exosome, MV and AB. TRAP staining was performed and number and size of OC were measured by ImageJ. Two-sided student's t-tests were performed. P=0.0005 (Exosomes vs MVs, n=5); p=0.0032 (Exosomes vs ABs, n=5); p= 0.0001 (Exosomes vs MVs, n=20 vs 10); p=0.0036 (Exosomes vs ABs, n=20 vs 10). Data are presented as mean values +/- SEM. Scale bar=100µm.

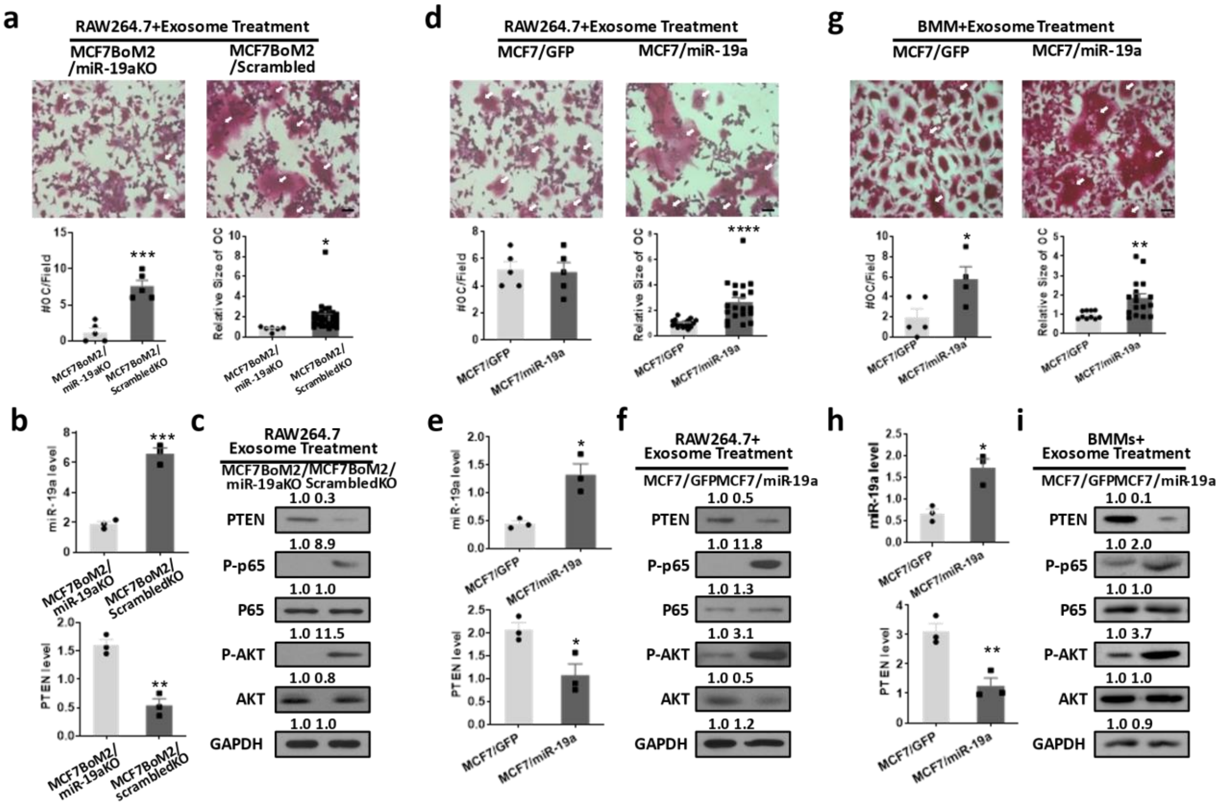

Supplementary Figure 6. Exosomal miR-19a targets PTEN/AKT pathway to promote osteoclastogenesis **(a)** TRAP staining was performed for RAW264.7 cells treated with exosomes from MCF7BoM2/miR-19aKO and MCF7BoM/ScrambledKO. The number of differentiated osteoclasts (p=0.0002, n=5) and the size of these cells (p=0.0392, n=6 vs 29) were measured. Two-sided student's t-tests were performed. Data are presented as mean values +/- SEM. Scale bar, 100µm. **(b)** Taqman PCR for miR-19a expression (p=0.0004, n=3) and SYBR Green qPCR for PTEN expression (p=0.0022, n=3) were performed for RAW264.7 cells that were treated with exosomes from MCF7BoM2/miR-19aKO and MCF7BoM/ScrambledKO. Two-sided student's t-tests were performed. Data are presented as mean values +/- SEM. **(c)** Western blot analysis was performed for PTEN, phospho-NF-κB P65, total NF-κB P65, phospho-AKT and total AKT expression in RAW264.7 cells that were treated with exosomes from MCF7BoM2/miR-19aKO and MCF7BoM/ScrambledKO. Band intensity was measure by ImageJ and the results were normalized to each control. **(d)** TRAP staining was performed for RAW264.7 cells that were treated with exosomes from MCF7/GFP and MCF7/miR-19a. Scale bar, 100µm. The number of differentiated osteoclasts (p=0.8327, n=5) and the size of these cells (p=0.000041, n=20 vs 20) were measured. Two-sided student's t-tests were performed. Data are presented as mean values +/- SEM. **(e)** Taqman PCR for miR-19a expression (p=0.0124, n=3) and SYBR Green qPCR for PTEN expression (p=0.0272, n=3) were performed for RAW264.7 cells that were treated with exosomes from MCF7/GFP and MCF7/miR-19a. Two-sided student's t-tests were performed. Data are presented as mean values +/- SEM. **(f)** Western blot analysis was performed for PTEN, phospho-NF-κB P65, total NF-κB P65, phospho-AKT and total AKT expression in RAW264.7 cells that were treated with exosomes from MCF7/GFP and MCF7/miR-19a. Band intensity was measure by ImageJ and the results were normalized to each control.

(g) TRAP staining was performed for BMMS that were treated with exosomes from MCF7/GFP and MCF7/miR-19a. The number of differentiated osteoclast (p=0.0362, n=5 vs 4) and the size of these cells (p=0.0082, n=10 vs 17) were measured. Two-sided student's t-tests were performed. Data are presented as mean values +/- SEM. **(h)** Taqman PCR for miR-19a expression (p=0.0101, n=3) and SYBR Green qPCR for PTEN expression (p=0.0084, n=3) were performed for BMMs that were treated with exosomes from MCF7/GFP and MCF7/miR-19a. Scale bar, 100µm. Two-sided student's t-tests were performed. Data are presented as mean values +/- SEM. **(i)** Western blot analysis was performed for PTEN, phospho-NF-κB P65, total NF-κB P65, phospho-AKT and total AKT expression in BMMs cells that were treated with exosomes from MCF7/GFP and MCF7/miR-19a. Band intensity was measure by ImageJ and the results were normalized to each control.

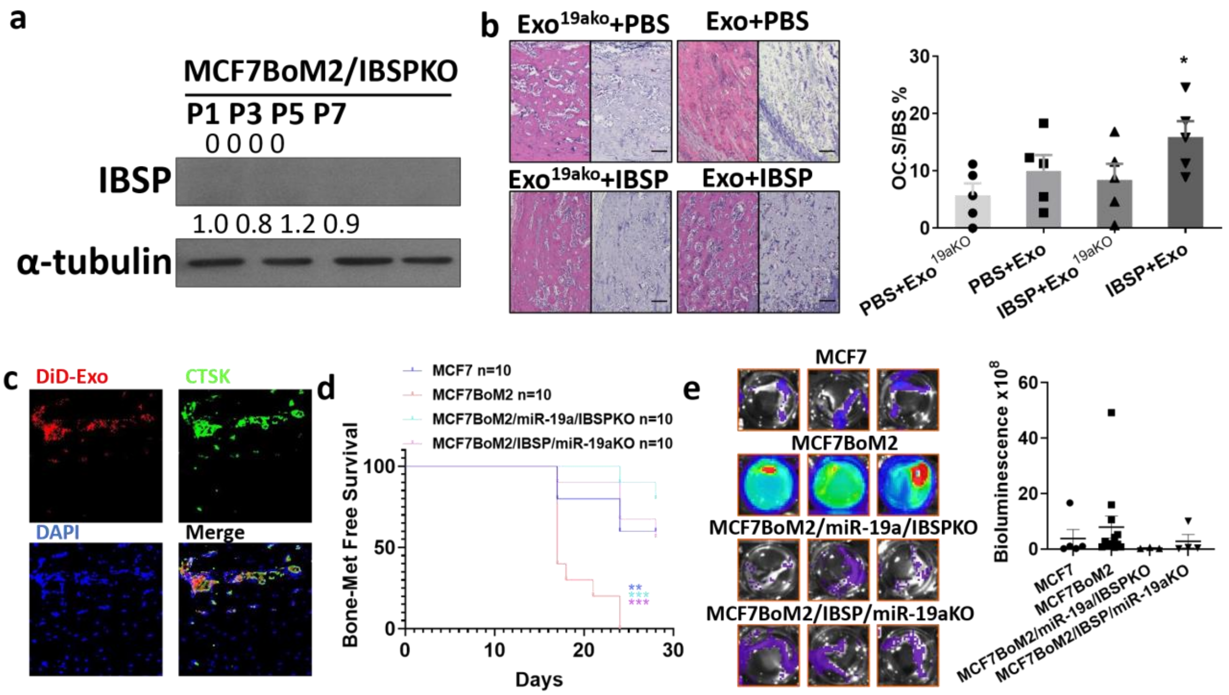

Supplementary Figure 7. IBSP and miR-19a cooperatively induce osteoclastogenesis

(a) The expression of IBSP in four different passages of MCF7BoM2/miR-19aKO was examined by western blot to confirm the stability of the knockout. The band was quantified by ImageJ and normalized to the first lane of α-tubulin. **(b)** The mice were treated with 2µg exosomes prepared from MCF7BoM2 and 0.5µg recombinant IBSP by injecting them into the tibias every two days. The exosomes from MCF7BoM2/miR-19aKO and PBS were used as controls. After two weeks, the bones were removed, and the TRAP staining of the bones was performed. The OC surface was quantified by ImageJ and the difference was analyzed by two-sided student's t-test. P=0.0181 (PBS+Exo^{19aKO} vs IBSP+Exo), p=0.2436 (PBS+Exo^{19aKO} vs PBS+Exo), p=0.4594 (PBS+Exo^{19aKO} vs IBSP+Exo^{19aKO}). N=5 in all groups. Data are presented as mean values +/- SEM. **(c)** The mice were treated with 2µg DiD-labeled exosomes prepared from MCF7BoM2 and 0.5µg recombinant IBSP by injecting them into the tibias every two days. After two weeks, the bones were extracted and stained with FITC-CTSK. The co-localization of OC and exosome was examined under the fluorescent microscope. **(d)** MCF7, MCF7BoM2, MCF7BoM2 cells with

ectopic expression of miR-19a and IBSP knockout, MCF7BoM2 cells with ectopic expression of IBSP and miR-19a knockout were intracardially injected into the mice. The growth of bone metastasis was monitored by IVIS Bioimager, and the Kaplan-Meier analysis and log-rank tests were performed for bone-metastasis free survival. P=0.0016 (MCF7 vs MCF7BoM2); p<0.0001 (MCF7BoM2 vs MCF7BoM2/miR-19a/IBSPKO); p=0.0004 (MCF7BoM2 vs MCF7BoM2/IBSP/miR-19aKO). **(e)** After one month, the legs of tumor-bearing mice were removed and their bioluminescence signals were measured. Data are presented as mean values +/- SEM.

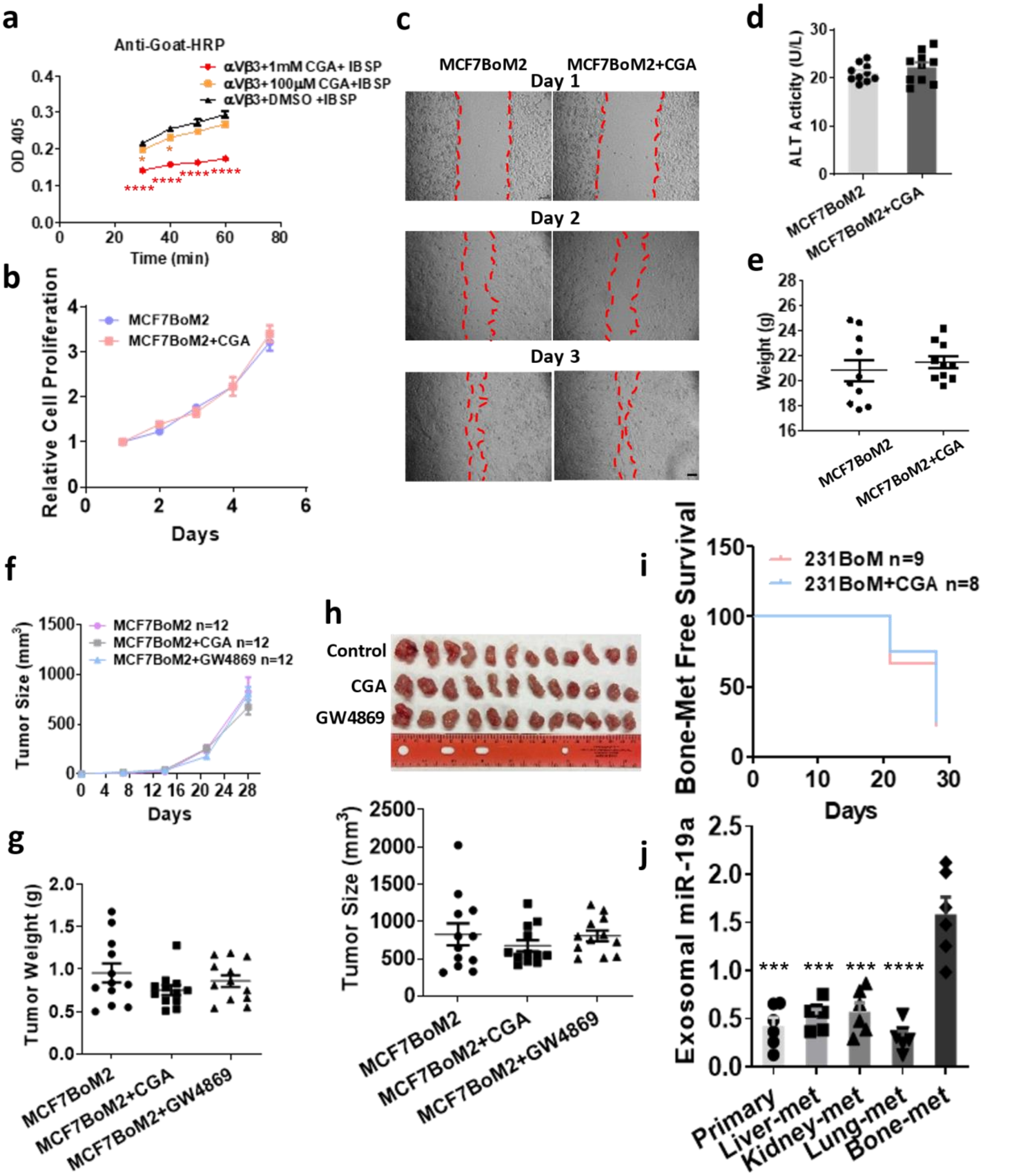

Supplementary Figure 8. CGA blocks blocks bone metastasis of ER$^+$ breast cancer cells

(a) In a competitive binding assay, integrin αVβ3 was seeded on the bottoms of 96-well plates, followed by adding IBSP in the presence or absence of a library of natural compounds. The bound IBSP was measured by colorimetric substrates of the horseradish peroxidase enzyme (HRP). The OD 405 was compared between CGA-treated groups and DMSO treated group. Two-sided student's t-tests were

performed. P=0.0424 (100µM vs DMSO, 30min), p=0.00001 (1mM vs DMSO, 30min), p=0.0499 (100µM vs DMSO, 40min), p=0.000014 (1mM vs DMSO, 40min), p=0.0723 (100µM vs DMSO, 50min), p=0.000014 (1mM vs DMSO, 50min), p=0.0751 (100µM vs DMSO, 60min), p=0.000013 (1mM vs DMSO, 60min). N=4 in all groups. Data are presented as mean values +/- SEM. **(b)** The effect of CGA on cell proliferation of MCF7BoM2 was measured by the MTS assay. Two-sided student's t-test was performed on day 5 and p=0.5105 (n=3 in both groups). Data are presented as mean values +/- SEM. **(c)** Effects of CGA on cell migration of MCF7BoM2 were assayed by wound healing assay. Scale bar, 100µm. **(d)** Effects of CGA treatment on liver function were evaluated by testing ALT (alanine aminotransferase) activity in mice. P=0.5332 and n=10 in each group. Data are presented as mean values +/- SEM. **(e)** Effects of CGA treatment on the bodyweight of mice were monitored. P=0.5332 and n=10 in each group. **(f)** MCF7BoM2 cells were injected into the mammary fat pad of mice. One group of the mice also received CGA treatment and another group received GW4869. The tumor growth was monitored and compared by measuring the size of the tumors. After four weeks, the tumors were removed and their weights **(g)** and sizes **(h)** were measured. (f) P=0.3647 (MCF7BoM2 vs MCF7BoM2+CGA), p=0.8996 (MCF7BoM2 vs MCF7BoM2+GW4869), and n=12. **(g)** P=0.162 (MCF7BoM2 vs MCF7BoM2+CGA), p=0.5005 (MCF7BoM2 vs MCF7BoM2+GW4869), and n=12. **(h)** P=0.3592 (MCF7BoM2 vs MCF7BoM2+CGA), p=0.9003 (MCF7BoM2 vs MCF7BoM2+GW4869), and n=12. (F-H) Data are presented as mean values +/- SEM. **(i)** Mice received 231BoM by intra-cardiac injection and they were treated with CGA. The growth of bone metastasis was monitored by IVIS Bioimager, and the Kaplan-Meier analysis was performed for bone-metastasis free survival (p=0842). **(j)** Primary T47D tumors, as well as organs with metastatic lesions from mice receiving T47D by intracardiac injection, were removed and cultured as explants for 48 hours in exosome-free media. After the culture, the conditioned media was collected from the explants and used to isolate exosomes. The amount of miR-19a expression in the exosomes was examined by PCR. Two-sided student's t-tests were performed. P=0.0002 (Primary vs Bone-met), p=0.0003 (Liver-met vs Bone-met), p=0.0005 (Kidney-met vs Bone-met), p=0.00005 (Lung-met vs Bone-met). N=6 in all groups. Data are presented as mean values +/- SEM.
