# Supplementary material for: Exosomal miR-19a and IBSP cooperate to induce osteolytic bone metastasis of estrogen receptor-positive breast cancer
Source: Nat Commun. 2021 Aug 31;12:5196. doi: 10.1038/s41467-021-25473-y (PMC8408156; doi:10.1038/s41467-021-25473-y)
Supplement: Supplementary file 2 — Description of Additional Supplementary Files [file 41467_2021_25473_MOESM2_ESM.docx]

**Description of Additional Supplementary Files**

File Name: Supplementary Data 1

Description: Primer sequences used for quantitative reverse transcription PCR are summarized in this table.

File Name: Supplementary Data 2

Description: Characteristics of the study population for investigating the exosomal miR-19a and free IBSP in the serum of breast cancer patients are summarized in this table.

File Name: Supplementary Data 3

Description: Detailed clinical information of each donor of the serum samples is summarized in this table.

File Name: Supplementary Data 4

Description: Detailed clinical information of each donor of the primary and metastatic tumor tissue, as well as the normal bone tissue, is summarized in this table.

File Name: Supplementary Data 5

Description: Serum samples are separated by treatment history. The effect of chemotherapy, endocrine therapy, and surgery on exosomal miR-19a and free IBSP levels is investigated. Samples from the ER^+^/Bone-met group have high miR-19a and IBSP levels, and they are unanimously collected from treatment-positive patients. To ensure a fair comparison, these samples are excluded from the analysis.
