# Supplementary material for: Exosomal miR-19a and IBSP cooperate to induce osteolytic bone metastasis of estrogen receptor-positive breast cancer
Source: Nat Commun. 2021 Aug 31;12:5196. doi: 10.1038/s41467-021-25473-y (PMC8408156; doi:10.1038/s41467-021-25473-y)
Supplement: Supplementary file 8 — Reporting Summary [file 41467_2021_25473_MOESM8_ESM.pdf]

Corresponding author(s): Kounosuke Watabe
Last updated by author(s): Jun 10, 2021

Reporting Summary

Nature Research wishes to improve the reproducibility of the work that we publish. This form provides structure for consistency and transparency in reporting. For further information on Nature Research policies, see our Editorial Policies and the Editorial Policy Checklist.

Statistics

For all statistical analyses, confirm that the following items are present in the figure legend, table legend, main text, or Methods section.

n/a	Confirmed	
☐	☒	The exact sample size (*n*) for each experimental group/condition, given as a discrete number and unit of measurement
☐	☒	A statement on whether measurements were taken from distinct samples or whether the same sample was measured repeatedly
☐	☒	The statistical test(s) used AND whether they are one- or two-sided<br>*Only common tests should be described solely by name; describe more complex techniques in the Methods section.*
☒	☐	A description of all covariates tested
☐	☒	A description of any assumptions or corrections, such as tests of normality and adjustment for multiple comparisons
☐	☒	A full description of the statistical parameters including central tendency (e.g. means) or other basic estimates (e.g. regression coefficient) AND variation (e.g. standard deviation) or associated estimates of uncertainty (e.g. confidence intervals)
☐	☒	For null hypothesis testing, the test statistic (e.g. *F*, *t*, *r*) with confidence intervals, effect sizes, degrees of freedom and *P* value noted<br>*Give P values as exact values whenever suitable.*
☒	☐	For Bayesian analysis, information on the choice of priors and Markov chain Monte Carlo settings
☒	☐	For hierarchical and complex designs, identification of the appropriate level for tests and full reporting of outcomes
☒	☐	Estimates of effect sizes (e.g. Cohen's *d*, Pearson's *r*), indicating how they were calculated

Our web collection on statistics for biologists contains articles on many of the points above.

Software and code

Policy information about availability of computer code

Data collection	Immunofluorescence, immunohistochemistry and cellular images were acquired using Keyence All-in-one fluorescence microscope (BZ-X700).Laser fluorescence scanning was performed with GenePix® 4000B Microarray Scanner. Time lapse was performed with Olympus IX-70 system. Electron microscopy was performed with FEI Tecnai BioTwin Transmission Electron Microscope (120 keV). Confocal Microscopy was performed with Zeiss LSCM 510 Laser Scanning Confocal Microscope. Bioluminescence was acquired using Xenogen IVIS imaging system (Caliper Life Science). Immunoblotting images were captured by Amersham Imager 600 as well as X-ray film processor. Quantitative PCR was performed with CFX Connect Real Time PCR system (Bio-rad). Absorbance of light was measured by FilterMax F5 (Molecular Devices). Flow cytometry was performed with BD FACS Canto II Analyzer. X-ray images of mice was acquired through Faxitron Multifocus 10x15 Digital Radiography System.
Data analysis	Bioluminescence data was analyzed using Living Image (Caliper Life Science) version 4.7.3 software and Aura software (Spectral Instruments Imaging, LLC) version 3.2. ImageJ (FIJI, 1.52n) was used for image quantification analysis. Statistical analysis was performed by GraphPad Prism (version 8.0). Cell migration was analyzed with Chemotaxis and Migration Tool (Version 2.0, IBIDI).

For manuscripts utilizing custom algorithms or software that are central to the research but not yet described in published literature, software must be made available to editors and reviewers. We strongly encourage code deposition in a community repository (e.g. GitHub). See the Nature Research guidelines for submitting code & software for further information.

Data

Policy information about availability of data

All manuscripts must include a data availability statement. This statement should provide the following information, where applicable:

- Accession codes, unique identifiers, or web links for publicly available datasets
- A list of figures that have associated raw data
- A description of any restrictions on data availability

CEL and expression files associated with the GeneChip miRNA array in this study are deposited at GEO (https://www.ncbi.nlm.nih.gov/geo/) with the accession number GSE176105. The GEO dataset (GSE41922, GSE22220, GSE56493, GSE2034), TCGA referenced during the study are available in a public repository from https://www.ncbi.nlm.nih.gov/gds and https://portal.gdc.cancer.gov/ websites. The source data underlying Figs 1-7and Supplementary Figs 1-8 are provided as a Source Data file. All the other data supporting the findings of this study are available within the article and its supplementary information files and from the corresponding author upon reasonable request. A reporting summary for this article is available as a Supplementary Information file.

Field-specific reporting

Please select the one below that is the best fit for your research. If you are not sure, read the appropriate sections before making your selection.

☒ Life sciences ☐ Behavioural & social sciences ☐ Ecological, evolutionary & environmental sciences

For a reference copy of the document with all sections, see nature.com/documents/nr-reporting-summary-flat.pdf

Life sciences study design

All studies must disclose on these points even when the disclosure is negative.

Sample size	Sample size was calculated based on previous experiment done (Liu Y, 2019; Xing F, 2018). Using power analysis, the anticipated means and standard error of means were included to determine the sample size that is expected to yield a power of approximately 80 percent using a p-value of 0.05.
Data exclusions	No data were excluded in our studies.
Replication	Numbers of the experimental replication or the experiments that were performed independently for each specific result were indicated in the Figure Legends.
Randomization	For cell experiments, all cells in each experiment were from the same pool of parental cells. All mice were age- and sex-matched (female mice) and then randomized into different experimental groups. All animals were maintained in the same environment and handled by the same procedure.
Blinding	For data collected by objective instruments, such as plate readers, qPCR cyclers, microscopy software, flow cytometers, animal IVIS systems, and western blotting, the investigators were not blinded to group allocation during data collection. However, investigator bias is not considered to contribute to the data because the investigator was blinded at the time of data analysis. Laboratory personnel were blind to animal randomization for drug treatment, which was performed by the PI. However, laboratory personnel could not be blinded during the experiment as they needed to know which groups to treat with which drugs. However, the laboratory personnel was blinded during the data analysis from each individual mice. Data analyses was performed by a biostatistician who was blinded to experimental groups.

Reporting for specific materials, systems and methods

We require information from authors about some types of materials, experimental systems and methods used in many studies. Here, indicate whether each material, system or method listed is relevant to your study. If you are not sure if a list item applies to your research, read the appropriate section before selecting a response.

Materials & experimental systems

n/a	Involved in the study	
☐	☒	Antibodies
☐	☒	Eukaryotic cell lines
☒	☐	Palaeontology and archaeology
☐	☒	Animals and other organisms
☐	☒	Human research participants
☒	☐	Clinical data
☒	☐	Dual use research of concern

Methods

n/a	Involved in the study	
☒	☐	ChIP-seq
☐	☒	Flow cytometry
☒	☐	MRI-based neuroimaging

Antibodies

Antibodies used

Rabbit anti-IBSP (Invitrogen, PA5-50633)
Goat anti-IBSP (R&D SYSTEMS, AF4014)
Normal Goat IgG Control (R&D SYSTEMS, AB-108-C)
Rabbit IgG Isotype Control (Invitrogen, 02-6102)
Rabbit anti-Phospho-NF-κB p65 (Ser536) (Cell Signaling Technology, clone 93H1, #3033)
Rabbit anti-E-cadherin (Cell Signaling Technology, clone 24E10, #3195)
Rabbit anti-NF-κB p65 (Cell Signaling Technology, #8242)
Rabbit anti-α-Tubulin (Cell Signaling Technology, #2144)
Rabbit anti-Phospho-Akt (Ser473) (Cell Signaling Technology, clone D9E, #4060)
Rabbit anti-Akt (Cell Signaling Technology, #9272)
Mouse anti-HSP70 (Invotrogen, Clone 5A5, #MA3-007)
Mouse anti-PTEN (Santa Cruz Biotechnology, clone A2B1, sc-7974)
Mouse anti-CD63 (Santa Cruz Biotechnology, clone MX-49.129.5, sc-5275)
Mouse anti-TSG101 (Santa Cruz Biotechnology, clone C-2, sc-7964)
Mouse anti-GRP94 (Santa Cruz Biotechnology, clone H-10, sc-393402)
Mouse anti-α-actinin-4 (Santa Cruz Biotechnology, clone G-4, sc-390205)
Mouse anti-Calregulin (Santa Cruz Biotechnology, clone A-9, sc-166837)
Rabbit anti-GAPDH (Cell Signaling Technology, clone D16H11, #5174)
Horseradish peroxidase-conjugated anti-mouse IgG (Cell Signaling Technology, #7076) or anti-rabbit IgG (Bio-Rad, #1706515)
Mouse IgG kappa binding protein m-IgGκ BP-HRP (Santa Cruz Biotechnology, sc-516102)
Donkey anti-Goat IgG (H+L) Secondary Antibody, HRP (Invitrogen, A15999)
Donkey anti-Goat IgG (H+L) Secondary Antibody, AP (Invitrogen, A16002)
Mouse Control IgG1 (InvivoGen, mabg1-ctrlm)
Mouse Control IgG2a (InvivoGen, mabg2a-ctrlm)
Mouse Control IgG2b (InvivoGen, mabg2b-ctrlm)

Validation

The following antibodies were quality-checked and validated based on the information provided on the manufacturers' websites:
Rabbit anti-IBSP (Invitrogen, PA5-50633): https://www.thermofisher.com/antibody/product/IBSP-Antibody-Polyclonal/PA5-50633
Goat anti-IBSP (R&D SYSTEMS, AF4014): https://www.rndsystems.com/products/human-ibsp-sialoprotein-ii-antibody_af4014
Normal Goat IgG Control (R&D SYSTEMS, AB-108-C): https://www.rndsystems.com/products/normal-goat-igg-control_ab-108-c
Rabbit IgG Isotype Control (Invitrogen, 02-6102): https://www.thermofisher.com/antibody/product/Rabbit-IgG-Isotype-Control/02-6102
Rabbit anti-Phospho-NF-κB p65 (Ser536) (Cell Signaling Technology, clone 93H1, #3033): https://www.cellsignal.com/products/primary-antibodies/phospho-nf-kb-p65-ser536-93h1-rabbit-mab/3033
Rabbit anti-E-cadherin (Cell Signaling Technology, clone 24E10, #3195): https://www.cellsignal.com/products/primary-antibodies/e-cadherin-24e10-rabbit-mab/3195
Rabbit anti-NF-κB p65 (Cell Signaling Technology, #8242): https://www.cellsignal.com/products/primary-antibodies/nf-kb-p65-d14e12-xp-rabbit-mab/8242
Rabbit anti-α-Tubulin (Cell Signaling Technology, #2144): https://www.cellsignal.com//products/primary-antibodies/a-tubulin-antibody/2144
Rabbit anti-Phospho-Akt (Ser473) (Cell Signaling Technology, clone D9E, #4060): https://www.cellsignal.com//products/primary-antibodies/phospho-akt-ser473-d9e-xp-rabbit-mab/4060
Rabbit anti-Akt (Cell Signaling Technology, #9272): https://www.cellsignal.com//products/primary-antibodies/akt-antibody/9272
Mouse anti-HSP70 (Invotrogen, Clone 5A5, #MA3-007): https://www.thermofisher.com/antibody/product/HSP70-Antibody-clone-5A5-Monoclonal/MA3-007
Mouse anti-PTEN (Santa Cruz Biotechnology, clone A2B1, sc-7974): https://www.scbt.com/p/pten-antibody-a2b1
Mouse anti-CD63 (Santa Cruz Biotechnology, clone MX-49.129.5, sc-5275): https://www.scbt.com/p/cd63-antibody-mx-49-129-5
Mouse anti-TSG101 (Santa Cruz Biotechnology, clone C-2, sc-7964): https://www.scbt.com/p/tsg-101-antibody-c-2
Mouse anti-GRP94 (Santa Cruz Biotechnology, clone H-10, sc-393402): https://www.scbt.com/p/grp-94-antibody-h-10
Mouse anti-α-actinin-4 (Santa Cruz Biotechnology, clone G-4, sc-390205): https://www.scbt.com/p/alpha-actinin-4-antibody-g-4
Mouse anti-Calregulin (Santa Cruz Biotechnology, clone A-9, sc-166837): https://www.scbt.com/p/calregulin-antibody-a-9
Rabbit anti-GAPDH (Cell Signaling Technology, clone D16H11, #5174): https://www.cellsignal.com/products/primary-antibodies/gapdh-d16h11-xp-rabbit-mab/5174
HRP-conjugated anti-mouse IgG (Cell Signaling Technology, #7076): https://www.cellsignal.com/products/secondary-antibodies/anti-mouse-igg-hrp-linked-antibody/7076
HRP-conjugated anti-rabbit IgG (Bio-Rad, #1706515) : https://www.bio-rad.com/en-us/sku/1706515-goat-anti-rabbit-igg-h-l-hrp-conjugate?ID=1706515
Mouse IgG kappa binding protein m-IgGκ BP-HRP (Santa Cruz Biotechnology, sc-516102): https://www.scbt.com/p/m-igg-kappa-bp-hrp
Donkey anti-Goat IgG (H+L) Secondary Antibody, HRP (Invitrogen, A15999): https://www.thermofisher.com/antibody/product/Donkey-anti-Goat-IgG-H-L-Secondary-Antibody-Polyclonal/A15999
Donkey anti-Goat IgG (H+L) Secondary Antibody, AP (Invitrogen, A16002): https://www.thermofisher.com/antibody/product/Donkey-anti-Goat-IgG-H-L-Secondary-Antibody-Polyclonal/A16002

Eukaryotic cell lines

Policy information about cell lines

Cell line source(s)	Breast carcinoma cell lines MDA-MB-231, MCF7, T47D, mouse monocyte cell line RAW264.7 were obtained from American Type Culture Collection (ATCC). MCF7-BoM2 and 231BoM-1833, the bone-metastatic derivatives of MCF7 and MDA-MB-231, were kindly provided by Dr. Joan Massagué (Memorial Sloan-Kettering Cancer Center).
Authentication	MDA-MB-231, MCF7, T47D and RAW264.7 were authenticated from ATCC by tests including: post-freeze viability, growth properties, morphology, COI assay, STR analysis.MCF7-BoM2 and 231BoM-1833, the bone-metastatic derivatives of MCF7 and MDA-MB-231 isolated from mice, were authenticated by PCR assays with species-specific primers.
Mycoplasma contamination	All cell lines used in this study were tested for mycoplasma contamination by PCR method. All cell lines used in this study were negative for mycoplasma.
Commonly misidentified lines (See ICLAC register)	None of cell lines used in this study are listed by ICLAC.

Animals and other organisms

Policy information about studies involving animals; ARRIVE guidelines recommended for reporting animal research

Laboratory animals	Athymic nude mice (female, all 5-6 weeks old at the beginning of each experiment) and C57BL/6 mice (female, all 5 weeks old at the beginning of each experiment), were housed in the Unit for Laboratory Animal Medicine at the Wake Forest Baptist Medical Center in compliance with the Institutional Animal Care and Use Committee regulations. Housing conditions at the Wake Forest Baptist Medical Center: Mice were maintained in a specific pathogen free unit on a 12hr light: 12hr dark cycle. The animal rooms are provided with 100% fresh, HEPA filtered air at 10-15 air changes per hour. Room temperatures are controlled by reheat units within each room, and are maintained within the range of 70°F ± 2° F. The Humidity levels are controlled globally, and it is maintained between 30-70%.
Wild animals	The study did not involve wild animals.
Field-collected samples	No field-collected samples were used in this study.
Ethics oversight	All experiments were reviewed and approved by the Institutional Animal Care and Use Committee (IACUC) of the Wake Forest Baptist Medical Center.

Note that full information on the approval of the study protocol must also be provided in the manuscript.

Human research participants

Policy information about studies involving human research participants

Population characteristics	Human biospecimens were collected retrospectively. Population characteristics including patient age, race, diagnosis, metastasis, receptor status and treatment history were retrieved from medical records, and summarized in table 1 and supplementary data 2.
Recruitment	No recruitment was involved. Human biospecimens were collected retrospectively from Tumor Tissue and Pathology Shared Resource (TTPSR) at Wake Forest Baptist Comprehensive Cancer Center (WFBCCC) and Cooperative Human Tissue Network.
Ethics oversight	All human biospecimens were collected under the IRB (Institute Review Board) approved protocol IRB00031311 at Wake Forest Baptist Medical Center.

Note that full information on the approval of the study protocol must also be provided in the manuscript.
